# A Guide to In Silico Drug Design

**DOI:** 10.3390/pharmaceutics15010049

**Published:** 2022-12-23

**Authors:** Yiqun Chang, Bryson A. Hawkins, Jonathan J. Du, Paul W. Groundwater, David E. Hibbs, Felcia Lai

**Affiliations:** 1 Sydney Pharmacy School, Faculty of Medicine and Health, The University of Sydney, Camperdown, NSW 2006, Australia; 2 Department of Biochemistry, Emory University School of Medicine, Atlanta, GA 30322, USA

**Keywords:** drug discovery, computer-aided drug design, *in silico* drug design

## Abstract

The drug discovery process is a rocky path that is full of challenges, with the result that very few candidates progress from hit compound to a commercially available product, often due to factors, such as poor binding affinity, off-target effects, or physicochemical properties, such as solubility or stability. This process is further complicated by high research and development costs and time requirements. It is thus important to optimise every step of the process in order to maximise the chances of success. As a result of the recent advancements in computer power and technology, computer-aided drug design (CADD) has become an integral part of modern drug discovery to guide and accelerate the process. In this review, we present an overview of the important CADD methods and applications, such as *in silico* structure prediction, refinement, modelling and target validation, that are commonly used in this area.

## 1. Introduction

New drugs with better efficacy and reduced toxicity are always in high demand, however the process of drug discovery and development is costly and time consuming and presents a number of challenges. The pitfalls of target validation and hit identification aside, a high failure rate is often observed in clinical trials due to poor pharmacokinetics, poor efficacy, and high toxicity [1,2]. A study conducted by Wong et al. that analysed 406,038 trials from January 2000 to October 2015 showed that the probability of success for all drugs (marketed and in development) was only 13.8% [3]. In 2016, DiMasi and colleagues [4] estimated a research and development (R&D) cost for a new drug of USD $2.8 billion based upon data for 106 randomly selected new drugs developed by 10 pharmaceutical companies. The average time taken from synthesis to first human testing was estimated to be approximately 2.6 years (31.2 months) and cost approximately USD $430 million, and from the start of a clinical testing to submission with the FDA was 6 to 7 years (80.8 months). In comparison to a study conducted by the same author in 2003, the R&D cost for a new drug had increased drastically by more than two-fold (from USD $1.2 billion) [5]. A possible reason for the increase in R&D cost is that regulators, such as the FDA have become more risk averse, tightening safety requirements, leading to higher failure rates in trials and increased costs for drug development. It is therefore important to optimise every aspect of the R&D process in order to maximise the chances of success.

**Figure 1:**
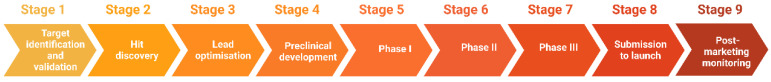
Stages of drug discovery and development.

The process of drug discovery starts with target identification, followed by target validation, hit discovery, lead optimisation, and preclinical/clinical development. If successful, a drug candidate progresses to the development stage, where it passes through different phases of clinical trials and eventually submission for approval to launch on the market (Figure 1) [6].

Briefly, drug targets can be identified using methods, such as data-mining [7], phenotype screening [8,9], and bioinformatics (e.g., epigenetic, genomic, transcriptomic, and proteomic methods) [10]. Potential targets must then be validated to determine whether they are rate limiting for the disease’s progression or induction. Establishing a strong link between the target and disease builds up confidence in the scientific hypothesis and thus greater success and efficiency in later stages of the drug discovery process [11,12].

Once the targets are identified and validated, compound screening assays are carried out to discover novel hit compounds (hit-to-lead). There are various strategies that can be used in this screening, involving physical methods, such as mass spectrometry [13], fragment screening [14,15], nuclear magnetic resonance (NMR) screening [16], DNA encoded chemical libraries [17], high throughput screening (HTS) (such as protein or cells) [18] or *in silico* methods, such as virtual screening (VS) [19].

After hit compounds are identified, properties, such as absorption, distribution, metabolism, excretion (ADME), and toxicity should be considered and optimised early in the drug discovery process. Unfavourable pharmacokinetic and toxicity profile of a drug candidate is one of the hurdles that often leads to failure in the clinical trials [20].

Although physical and computational screening techniques are distinct in nature, they are often integrated in the drug discovery process to complement each other and maximise the potential of the screening results [21].

**Figure 2:**
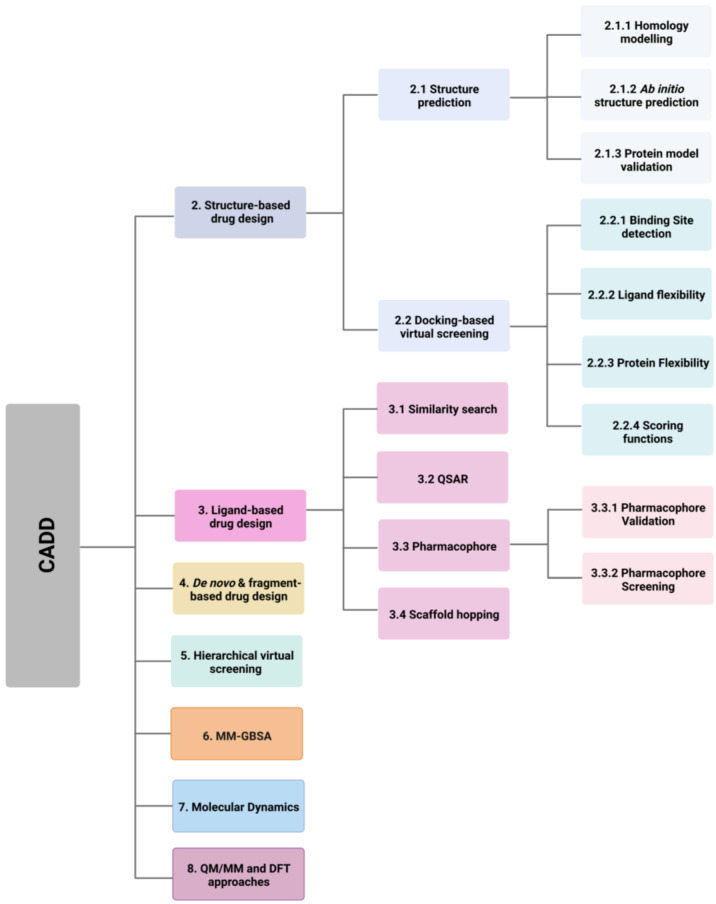
Various *in silico* techniques used in the drug design and discovery process discussed in this review. (Abbreviations: CADD: computer-aided drug design; DFT: density functional theory; MM: molecular mechanical; MM-GBSA: molecular mechanics with generalised Born and surface area; QM: quantum mechanical; QSAR: quantitative structure activity relationship).

Computer-aided drug design (CADD) utilises this information and knowledge to screen for novel drug candidates. With the advancement in technology and computer power in recent years, CADD has proven to be a tool that reduces the time and resources required in the drug discovery pipeline. The aim of this review is to give an overview of the various *in silico* techniques that are used in the drug discovery process (Figure 2).

## 2. Structure-Based Drug Design

**Figure 3:**
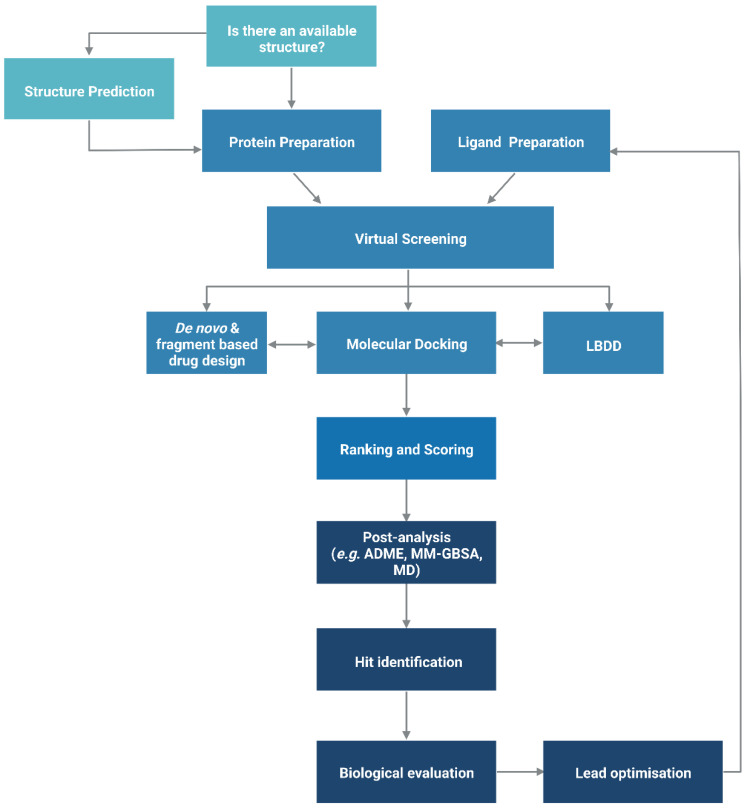
General workflow of molecular docking. The process begins with the preparation of the protein structure and ligand database separately, followed by molecular docking in which the ligands were ranked based on their binding pose and predicted binding affinity. (Abbreviations: LBDD: Ligand-based drug design; ADME: absorption, distribution, metabolism and excretion; MD: molecular dynamics; MM-GBSA: molecular mechanics with generalised Born and surface area).

The functionality of a protein is dependent upon its structure, and structure-based drug design (SBDD) relies on the 3D structural information of the target protein, which can be acquired from experimental methods, such as X-ray crystallography, NMR spectroscopy and cryo-electron microscopy (cryo-EM). The aim of SBDD is to predict the Gibbs free energy of binding (ΔG_bind_), the binding affinity of ligands to the binding site, by simulating the interactions between them. Some examples of SBDD include molecular dynamics (MD) simulations [22], molecular docking [23], fragment-based docking [24], and *de novo* drug design [25]. Figure 3 describes a general workflow of molecular docking that will be discussed in greater detail.

### 2.1. Protein Structure Prediction

The advancements in sequencing technology led to a steep increase in recorded genetic information thus rapidly widening the gap between the amounts of sequence and structural data available. As of May 2022, the UniprotKB/TrEMBL database contained over 231 million sequence entries, yet there are only approximate 193,000 structures recorded in the Protein Data Bank (PDB) [26,27]. To model the structures of those proteins where structural data is not available, homology (comparative) modelling or *ab initio* methods can be used.

#### 2.1.1. Homology Modelling

Homology modelling involves predicting the structure of a protein by aligning its sequence to a homologous protein that serves as a template for the construction of the model. The process can be broken down into three steps: (1) template identification, (2) sequence-template alignment, and (3) model construction.

Firstly, the protein sequence is obtained, either experimentally or from databases, such as the Universal Protein Resource (UniProt) [28], and this is followed by identifying modelling templates that have high sequence similarity and resolution by performing a BLAST [29] search against the Protein Data Bank [30]. PSI-BLAST [29] uses profile-based methods to identify patterns of residue conservation, which can be more useful and accurate than simply comparing raw sequences, as protein functions are predominately determined by the structural arrangement rather than the amino acid sequence. One of the biggest limitations of homology modelling is that it relies heavily upon the availabilities of suitable templates and accurate sequence alignment. A high sequence identity between the query protein and the template normally gives greater confidence in the homology model. Generally, a minimum of 30% sequence identity is considered to be a threshold for successful homology modelling, as approximately 20% of the residues are expected to be misaligned for sequence identities below 30%, leading to poor homology models. Alignment errors are less frequent when the sequence identity is above 40%, where approximately 90% of the main-chain atoms are likely to be modelled with a root-mean-square deviation (RMSD) of ~1 Å, and the majority of the structural differences occur at loops and in side-chain orientations [31].

**Figure 4:**
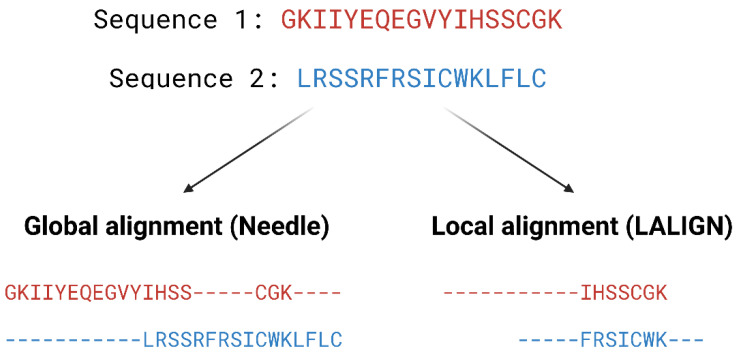
Example of global and local alignment using Needle [32] and LALIGN [32]. Global alignment aims to find the best alignment across the two entire length of sequences. Local alignment finds regions of high similarity in parts of the sequences.

Pairwise alignment methods are used when comparing two sequences and they are generally divided into two categories—global and local alignment (Figure 4). Global alignment aims to align the entire sequences and are most useful when sequences are closely related or of similar lengths. Tools such as EMBOSS Needle [32] and EMBOSS Stretcher [32] use the Needleman–Wunsch algorithm [33] to perform global alignment. In comparison to using a somewhat brute-force approach, the Needleman–Wunsch algorithm uses dynamic programming to find the best alignment by reducing the number of possible alignments that need to be considered and guarantees to find the best alignment. Dynamic programming aims to break a larger problem (the entire sequence) into smaller problems which are then solved optimally. The solutions to these smaller problems are then used to construct an optimal solution to the original problem [34]. The Needleman–Wunsch algorithm first builds a matrix that is subjected to a gap penalty (negative scores in first row and column), and the matrix is used to assign a score to every possible alignment (usually positive score for match, no score or penalty for mismatch and gaps). Once the cells in the matrix are filled in, traceback starts from the lower right towards the top left of the matrix to find the best alignment with the highest score.

Local alignment, on the other hand, aims to identify regions that share high sequence similarity, which is more useful when aligning sequences that are dissimilar or distantly related. EMBOSS water [32] and LALIGN [32] are tools that use the Smith–Waterman algorithm [35] for local alignment. The Smith–Waterman algorithm, such as the Needleman-Wunsch algorithm, uses dynamic programming to perform sequence alignment. However, there is no negative score assigned in this algorithm, and the first row and column are set to 0. Traceback begins with the matrix cell from the highest score and travels up/left until it reaches 0 to produce the highest scoring local alignment.

When searching for templates used for homology modelling, including multiple sequences will improve accuracy of the alignment in regions where there is a low sequence homology, hence multiple sequence alignment (MSA) is essential. The global alignment method for multiple sequences is generally too computationally expensive; modern MSA tools (e.g., ClustalW [36], T-Coffee [37] and MUSCLE [38]) commonly use a progressive alignment approach that combines global and/or local alignment methods, followed by the branching order of a guide tree. This technique aims to achieve a succession of pairwise alignments, first aligning the most similar sequences and then progressing to the next most similar sequence until the entire query set has been incorporated.

For example, MSA was used during the construction of the homology models for Alanine-Serine-Cysteine transporter (SLC1A5) by Garibsingh et al. At the time, there was limited structural information on SLC1A5 due to the lack of an experimentally determined structure of human SCL1 family proteins. Most of the knowledge on the human SLC1 family protein therefore came from the study of prokaryotic homologs, which share low sequence identity. Using the structural information of the recently solved human SLC1A3, Garibsingh et al. carried out a phylogenetic analysis by generating MSA of the human SCL1 family and its prokaryotic homologs using MUSCLE and Promals3D [39], and built two different conformations of SLC1A5 homology models for the design of SLC1A5 inhibitors [40].

Once the alignment is complete, the model can be constructed starting with the backbone, then loops and lastly side-chains. The polypeptide backbone of the protein is first created by copying the coordinates of the residues from the template to create the model backbone. Gaps between the alignment of the sequence and the template are then taken care of through insertions and deletions in the alignment. It is important to remodel gaps accurately, as any error introduced here, will be amplified in later stages, thus leading to structural changes that can be critical for protein functionality and protein–protein interactions. Loop modelling, via knowledge-based methods or energy-based methods, can be used to generate predictions of the conformations of the loop. Knowledge-based methods look for experimental data on loops with high sequence similarity to the target from databases, such as PDB, and then insert them into the model. Yang et al. used FREAD [41] to predict the structure of a missing loop and construct a model of a monoclonal antibody, Se155-4, to study its antibody–antigen interactions with *Salmonella Typhimurium* O polysaccharide [42]. On the other hand, energy-based methods predict protein folding using *ab initio* methods with scoring function optimisation. For example, the Rosetta Next-Generation Kinematic Closure protocol [43], which employs the *ab initio* method, was used in loop prediction calculations to construct parts of the leucine-rich repeat kinase 2 (LRRK2) model, as the homology model template had missing loop sections. Mutations in the catalytic domains of LRRK2 are associated with familial and sporadic Parkinson’s disease, yet little is known about its overall structure and the mutations, which alter LRRK2 function and enzymatic activities. Combining homology models with experimental constraints, Guaitoli and co-workers constructed the first structural model of the full length LRRK2 that includes domain engagement and contacts. The model provided insight into the roles that the different domains play in the pathogenesis of Parkinson’s disease and will serve as a basis for future drug design on LRRK2 [44].

Lastly, side-chains are built onto the backbone model according to the target sequence. Most side-chain types in proteins have a limited number of conformations (rotamers) and programs such as SCWRL [45] predict these in order to minimise the total potential energy. Upon completion, the model is optimised using molecular mechanics force fields to improve its quality.

A ligand-based approach can be utilised to further optimise homology models with low sequence identity between query sequence and structural template. Moro et al. first presented ligand-based homology modelling, also known as ligand-guided or ligand-supported homology modelling, as a tool to inspect G protein-coupled receptors (GPCRs) structural plasticity [46]. GPCRs comprise a superfamily of membrane proteins with over 800 members; they play a significant role in cellular signalling in the human body. As such, GPCRs are associated with numerous biological processes, making them important therapeutic targets [47]. Unfortunately, crystallisation of membrane proteins is known to be challenging, especially in the case of GCPRs, and there were few structural data of GPCRs available until the last decade.

Given that the GPCRs are a diverse family, additional optimisation is required to refine homology models built for those with low sequence identity to the structural template to increase the level of accuracy. In this approach, an initial homology model is first developed using the conventional method. Active ligands are then docked into the binding site for optimisation. The receptor is reorganised and refined based upon the ligand binding in order to better accommodate ligands with higher affinity. Moro et al. first introduced this approach to construct a homology model of the human A_3_ receptor based on the structure of bovine rhodopsin in 2006, the only known GPCR structure at the time. A set of structurally related class of pyrazolotriazolopyrimidines with known binding affinities was docked into a conventional rhodopsin-based homology model to induce receptor reorganisation [46].

The ligand-based homology modelling approach has been used extensively since then in studies of GPCRs, including serotonin receptors [48], dopamine receptors [49], cannabinoid receptors [50], neurokinin-1 receptor [51], γ-aminobutyric acid (GABA) receptor [52] and histamine H3 receptors [53].

#### 2.1.2. *Ab Initio* Protein Structure Prediction

Historically, the homology modelling approach has been the ‘go-to’ method when it comes to protein structure prediction because it is less computationally expensive and produces more accurate predictions. One of the biggest limitations, however, is that it relies on existing known structures, so that the prediction of more complex targets, such as membrane proteins with little known structural data, is almost impossible. Another solution to this problem is the use of template-free approach, also known as *ab initio* modelling, free modelling, or *de novo* modelling [54,55]. As the name implies, this approach predicts a protein structure from amino acid sequences without the use of a template. In addition, the *ab initio* approach can model protein complexes and provide information on complex formation and protein-protein interaction. This is significant as some proteins exist as oligomers and hence performing docking on monomeric structures may be ineffective [56]. The principle behind *ab initio* modelling is based on the thermodynamic hypothesis proposed by Anfinsen, which states that ‘the three-dimensional structure of a native protein in its normal physiological milieu is the one in which the Gibbs free energy of the whole system is lowest; that is that the native conformation is determined by the totality of the inter atomic interactions, and hence by the amino acid sequence, in a given environment [57].

*Ab initio* protein structure prediction is traditionally classified into two groups, physics-based and knowledge-based, although recent approaches tend to incorporate both. Purely physics-based methods such as ASTRO-FOLD [58,59] and UNRES [60] are independent of structural data and the interactions between atoms are modelled based on quantum mechanics. It is believed that all the information about the protein, including the folding process and its 3D structure, can be deduced from the linear amino acid sequence. This approach is often coupled with molecular dynamics refinement which also gives valuable insight into the protein folding process. The Critical Assessment of Methods of Protein Structure (CASP) is a biennial double-blinded structure prediction experiment that assesses the performance of various protein structure prediction methods. ASTRO-FOLD 2.0 successfully predicted a number of good quality structures that are comparable to the best model in CASP9 [59]. Unfortunately, one of the major drawbacks of pure physics-based approaches is that, due to the enormous amount of conformational space needed to cover, it is often accompanied with high computational cost and time requirement and is only feasible to predict the structures of small proteins.

Bowie and Eisenberg first proposed the idea of assembling short fragments derived from existing structures to form new tertiary structures in 1994 [61]. The idea behind this process is that the use of low-energy local structures from a fragment library provides confidence in local features as these structures are experimentally validated. Furthermore, significantly reduced computational resources are required as the conformational sampling space is reduced. Rosetta, one of the best-known knowledge-based programs, utilises a library of short fragments that represent a range of local structures by splicing 3D structures of known protein structures. The query sequence is then divided into short ‘sequence window’; the top fragments for each sequence window are identified, on the basis of factors, such as sequence similarity and secondary structure prediction for local backbone structures, and these fragments are assembled to build a pool of structures with favourable local and global interactions (known as decoys) via a Monte Carlo sampling algorithm [62]. During the assembly process, the representation of the structure is simplified (only includes the backbone atoms and a single centroid side-chain pseudo-atom) in order to sample the conformational space efficiently. It starts off with the protein in a fully extended conformation. A sequence window is selected and one of the top ranked fragments for this window is randomly selected to have its torsion angles replace those of the protein chain. The energy of the conformation is then evaluated by a course-grained energy function and the move accepted or rejected according to the Metropolis criterion. In the Metropolis criterion, a conformation with a lower energy than the previous one is accepted, whereas a conformation with a higher energy (less favourable) is kept based on the acceptance probability [63]. The whole process repeats until the whole 3D structure is generated. Following this, side-chains are constructed and structures are refined using an all-atom energy function to model the position of every atom in the structure and generate high resolution models [64]. Other knowledge-based *ab initio* approaches include I-TASSER [65] and QUARK [66].

Another method to improve the accuracy of *de novo* protein structure prediction is the use of co-evolutionary data for targets with many homologs. The structure of a protein is the key to its biological function, and through the evolutionary process, amino acids in direct physical contact, or in proximity, tend to co-evolve together in order to maintain these interactions and hence preserve the function of the protein. Furthermore, residues that have a high number of evolutionary constraints could indicate important functionalities. Based upon this principle, evolutionary and co-variation data that are obtained from databases such as Pfam [67] can be harnessed to predict residue contacts and protein folding [68]. This method works by performing MSA on a large and diverse set of homolog sequences to the query protein, information on amino acids pairs that co-evolve, also known as evolutionary couplings, are then extracted to determine the location of each residues [69].

The application of neural network-based deep learning approaches to integrate co-evolutionary information has revolutionised the technology used in protein structure prediction and made a huge impact. There are currently a few prediction approaches using deep learning methods to guide protein structure prediction, such as Raptor X [70], ProQ3D [71], D-I-TASSER [72], D-QUARK [72], and trRosetta [73]. The impact of using deep learning methods is showcased by AlphaFold, an Artificial Intelligence (AI) system developed by DeepMind and RoseTTAFold [74], a similar program built using a 3-track neural network from the Baker lab, which has taken the protein modelling community by storm in the two most recent CASPs, CASP13 and CASP14. In CASP13, Alphafold 1 [75] was placed first in the rankings with an average of Global Distance Test Total Score (GDT_TS) of 70%. The GDT_TS is a metric that corresponds to the accuracy of the backbone of the model, the higher the value, the higher the accuracy [76]. Subsequently in CASP14, the newer version, Alphafold 2, was placed first again and outperformed all other programs by a huge margin with a median GDT_TS of 92.4 over all categories [77]. Additionally, the updated version of trRosetta, RoseTTaFold, was ranked second and demonstrated a superior performance than AlphaFold 1 in CASP13, and that all top 10 ranking methods in CASP14 use deep learning-based approaches, signifying the progression in protein prediction accuracy. High accuracy models predicted by AlphaFold 2 are also published in AlphaFold Protein Structure Database (https://alphafold.ebi.ac.uk/, accessed on 7 May 2022), providing an extensive structural coverage of known protein sequences [78].

Knowledge-based methods, such as I-TASSER and QUARK were not tested in CASP14 [72], however variants of these approaches which integrated deep-learning into protein structure prediction algorithms ranked 8th and 9th, respectively. Physics-based methods, such as UNRES (previously described above), using 3 different approaches (UNRES-template, UNRES-contact and UNRES) achieved GDT_TS scores of 56.37, 39.3 and 29.2, respectively. These results ranked 32nd, 109th and 117th [77]. The large majority of the top ranking algorithms in CASP14 utilised deep learning approaches, further affirming the utility of deep learning in protein structure prediction approaches [72].

#### 2.1.3. Protein Model Validation

**Figure 5:**
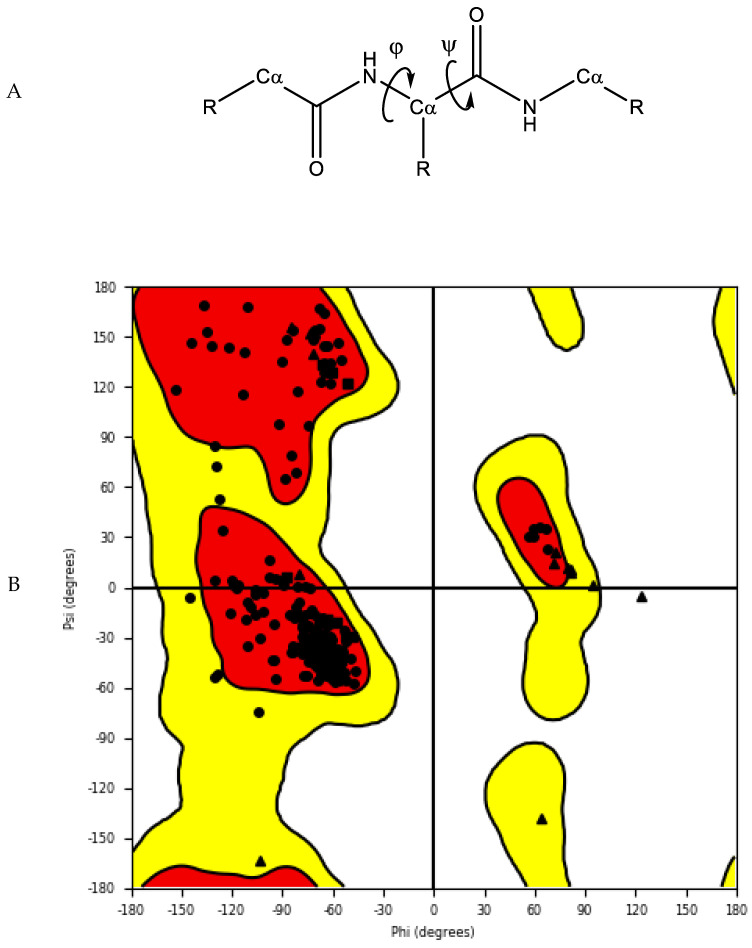
(**A**) Protein backbone with dihedral angles. (**B**) An example of a Ramachandran plot of crystal structure of human farnesyl pyrophosphate synthase (PDB ID: 4P0V) [91]. White: disallowed region; yellow: allowed region; red: favourable region.

The accuracy and quality of the predicted structures can be validated and verified using different methods. The stereochemistry of the model can be verified by analysing bond lengths, torsion angles and rotational angles with tools, such as WHATCHECK [79] and Ramachandran plots [80]. The Ramachandran plot examines the backbone dihedral angles ϕ and ψ, which represents the rotations made by N—Cα and Cα—C bond in the polypeptide chain, respectively (Figure 5). Torsion angles determine the conformation of each residue and the peptide chain; however, some angle combinations cause close contacts between atoms, leading to steric clashes. The Ramachandran plot determines which torsional angles of the peptide backbone are permitted, and thus assesses the quality of the model. Spatial features, such as 3D conformation and mean force statistical potentials, can be validated using Verify3D [81], which measures the compatibility of the model to its own amino acid sequence. Each residue in the model is evaluated by its environment, which is defined by the area of the residue that is buried, the fraction of side-chain area that is covered by polar atoms (oxygen and nitrogen) and the local secondary structure. Other structure validation tools include MolProbity [82,83], NQ-Flipper [84], Iris [85], SWISS-MODEL [86] and *Coot* [87,88,89]. In addition to *in silico* validation, experimental validation of the predicted complexes may also be used to aid selection of a model for future *in silico* studies. Cross-linking mass spectrometry (XL-MS) provides experimental distance constraints, which can be checked against the predicted models [90].

### 2.2. Docking-Based Virtual Screening

Docking-based virtual screening aims to discover new drugs by predicting binding modes of both ligand and receptor, studying their interaction patterns, and estimating binding affinity. Some examples of the many docking programs include AutoDock [92], GOLD [93], Glide [94,95], SwissDock [96], DockThor [97], CB-Dock [98] and Molecular Operating Environment (MOE) [99] (Table 1). Due to limitations of X-ray crystallography and NMR spectroscopy, experimentally derived structures often have problems, such as missing hydrogen atoms, incomplete side-chains and loops, ambiguous protonation states and flipped residues. It is therefore essential to prepare the 3D structures accordingly in order to fix these issues before the docking process [100].

The three main goals of molecular docking are: (1) pose prediction to envisage how a ligand may bind to the receptor, (2) virtual screening to search for novel drug candidates from small molecule libraries and (3) binding affinity prediction using scoring functions to estimate the binding affinity of ligands to the receptor [101]. Search algorithms and scoring functions are essential components for molecular docking programs.

A good search algorithm should explore all possible binding modes, and this can be a challenging task. The concept of molecular docking originated from the ‘lock and key’ model proposed by Emil Fischer [102], and early docking programs treated both the protein and ligands as rigid bodies. It was known that protein and ligands are both dynamic entities and that their conformations play an important role in ligand–receptor binding and protein functions, but historically this was too computationally expensive to implement. Modern docking programs treat both protein and ligand with varying degrees of flexibility in order to address this issue.

#### 2.2.1. Binding Site Detection

In docking-based virtual screening, the location of the binding site within the protein must be identified. Most of the protein structures in the PDB are ligand-bound (*holo*) structures, which defines the binding pocket and provides us with its geometries. In cases where only ligand-free (*apo*) structures available, there are traditionally three main types of method to identify potential druggable binding sites. Template-based methods such as *firestar* [103], 3DLigandSite [104] and Libra [105,106] utilise protein sequences to locate residues that are conserved and important for binding. Geometry-based methods, such as CurPocket [98], Surfnet [107] and SiteMap [108,109], search for clefts and pockets based on the size and depths of these cavities. Energy-based methods such as FTMap [110] and Q-SiteFinder [111] locate sites on the surface of a protein that are energetically favourable for binding. Hybrid methods, such as ConCavity [112] and MPLs-Pred [113], as well as machine-learning methods, such as DeepSite [114], Kalasanty [115], and DeepCSeqSite [116] are some of the newer approaches that are under rapid development in recent years.

Beyond locating the orthosteric binding site, these tools are also valuable in identifying potential allosteric binding sites to modulate protein function, hot spots on protein surface to alter protein–protein interactions and also analysing known binding sites to design better molecules that complement the binding pocket. Furthermore, proteins are dynamic systems, and their conformations may change upon ligand binding. Hidden binding pockets, known as cryptic pockets, which are not present in a ligand-free structure, can result from conformational changes upon ligand binding. Detection of cryptic pockets can be a solution to target proteins that were previously considered to be undruggable due to the lack of druggable pockets [117,118].

In addition to the location of the binding site, the evaluation of its potential druggability is equally important. Druggability is the likelihood of being able to modulate a target with a small molecule drug [119]. It can be evaluated on the basis of target information and association, such as protein sequence similarity or genomic information [120]. However, this approach only works for well-studied protein families and homologous proteins may not necessarily bind to structurally similar molecules [121].

Various efforts have been made to evaluate druggability using structure-based approaches. Cheng et al. developed the MAP_POD_ score, one of the first methods to evaluate druggability, using a physics-based method. MAP_POD_ model is a binding free energy model combined with curvature and hydrophobic surface area to estimate the maximal achievable affinity for passively absorbed drugs [119]. Halgren developed Dscore, which is a weighted sum of size, enclosure and hydrophobicity [108,109,122]. Other methods to predict druggability include Drug-like Density (DLID) [123], DrugPred [124], DoGSiteScorer [125], FTMap [126] and PockDrug [127].

DoGSiteScorer is a webserver that supports the prediction of potential pockets, characterisation and the druggability estimation. The algorithm first maps a rectangular grid onto the protein; grid points are labelled as either free or occupied depending on whether they lie within the vdW radius of any protein atom. Free grid points are merged to form pockets and subpockets, and neighbouring subpockets are then merged to form pockets. A 3D Difference of Gaussian (DoG) filter is then applied to identify pockets that are favourable to accommodate a ligand. These pockets are characterised global and local descriptors, such as pocket volume, surface, depth, ellipsoidal shape, types of amino acids, presence of metal ions, lipophilic surface, overall hydrophobicity ratio, distances between functional group atoms and many more [125,128].

To predict druggability, a machine learning technique (support vector machine model) trained on a set of known druggable proteins is used to identify druggable pockets based on a subset of these descriptors and to provide a druggability score between 0 to 1, where the higher the score the more druggable is the pocket. A SimpleScore, a linear regression based on size, enclosure and hydrophobicity, is also available to predict druggability [129].

Michel and co-workers used DoGSite, along with FTMap, CryptoSite, as well as SiteMap to predict ligand binding pockets and evaluate druggability of the nucleoside diphosphates attached to sequence-x (NUDIX) hydrolase protein family. Using a dual druggability assessment approach, the authors identified several proteins that are druggable out of the 22 that were studied. This *in silico* data was also found to correlate well with experimental results [130].

Sitemap locates binding sites by placing ‘site points’ around the protein and each site point is analysed for the proximity to the protein surface and solvent exposure. Site points that fulfil the criteria and are within a given distance of each other are combined into subsites, then subsites that have a relatively small gap between them in a solvent-exposed region are merged to form sites. Distance-field and van der Waals (vdW) grids are then generated to characterise the binding site into three basic regions: hydrophobic, hydrophilic (further separates into H-bond donor, acceptor, and metal-binding region) and neither. Sitemap also evaluates the potential binding sites and computes various properties such as size of the site measured by number of site points, exposure to solvent, degree of enclosure by protein, contact of site points with the protein, hydrophobic and hydrophilic character of the site, and the degree to which a ligand can donate hydrogen bonds. These properties contribute to the calculation of the SiteScore (to distinguish drug-binding and non-drug binding sites) and Dscore (druggability score), which helps to recognise druggable binding sites for virtual screening [108,109].

**Figure 6:**
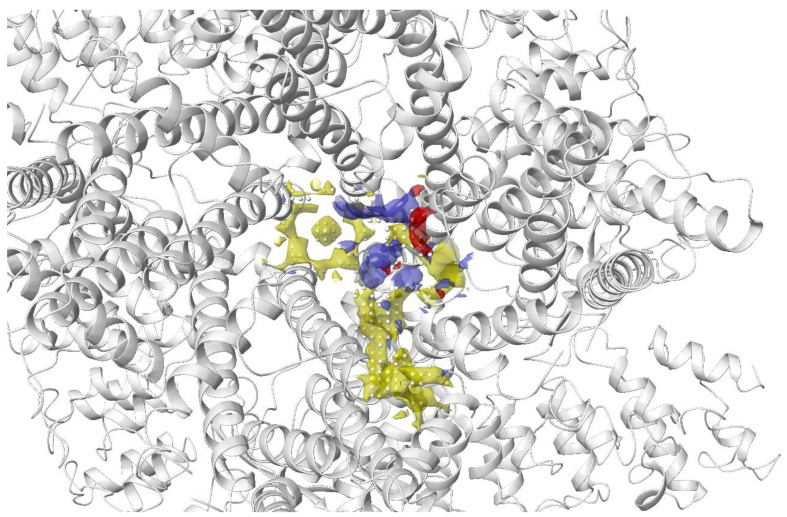
Binding site of TRPV4 detected using Sitemap by Doñate-Macian et al. [131]. Yellow: hydrophobic region; blue: H-bond donor region; red: H-bond acceptor region; white sphere: site point.

The transient receptor potential vanilloid 4 (TRPV4) is a widely expressed non-selective cation channel involved in various pathological conditions. Despite the availability of several TRPV4 inhibitors, the binding pocket of TRPV4 and the mechanism of action was not well understood. Doñate-Macian and coworkers used Sitemap to search and assess the binding pocket for one of the known TRPV inhibitors HC067047 based on the crystal structure of Xenopus TRPV4 (Figure 6). This group also further characterised the binding pocket and inhibitor–protein binding interactions with the aid of molecular docking, molecular dynamics and mutagenesis studies. The information was then employed to run a structure-based virtual screening to discover novel TRPV4 inhibitors [131].

#### 2.2.2. Ligand Flexibility

Ligand structures for virtual screening can be obtained from small molecule databases, which are free (e.g., ZINC [132], DrugBank [133] and Pubchem [134]) or commercial (e.g., Maybridge, ChemBridge and Enamine). Conformational sampling of ligands can be performed in several ways. Systematic search generates all possible ligand conformations by exploring all degrees of freedom of the ligand [135]. Carrying out a systematic search using a brute-force approach (exhaustive search) can easily overwhelm the computing power, especially for molecules with many rotatable bonds and therefore rule-based methods have been the more favoured approaches in recent years. Rule-based methods, such as the incremental construction algorithm (also known as anchor and grow method), generate conformations based on known structural preferences of compounds by limiting the conformational space that is being explored. Usually, a knowledge base of allowed torsion angles and ring conformations (e.g., data from the PDB), and possibly a library of 3D fragment conformations, is used to guide the sampling [136,137]. These break the molecule into fragments that are docked into different regions of the receptor. The fragments are then reassembled together to construct a molecule in a low energy conformation.

Conformer generator OMEGA [138] employs a prebuilt library of fragments as well as a knowledge base of torsion angles to generate a large set of conformations, which are sampled by geometric and energy criteria to eliminate conformers with internal clashes. Likewise, ConfGen [139] divides ligands into a core region and peripheral rotamer groups. The core conformation is first generated using a template library, followed by the calculation of the potential energy of rotatable bonds with the torsional term of the OPLS force field, and lastly positioning peripheral groups in their lowest energy forms. To eliminate undesirable conformations or to limit the number of conformations, filtering approaches are applied. Conformations that are too similar are removed based on an energy filter, RMSD, and dihedral angles involving polar hydrogen atoms. Compact conformers are also removed by an empirically derived heuristic scoring method [94,139].

On the other hand, a stochastic search randomly changes the degrees of freedom of the ligand at each step and the change is either accepted or rejected according to a probabilistic criterion such as the Metropolis criterion [140]. Sampling of conformational space can be performed using different techniques in a stochastic search, including Monte Carlo (MC) sampling [62], distance geometry sampling [141] and genetic algorithm-based sampling [142,143]. Balloon [142], a free conformer generator, uses distance geometry to generate an initial conformer for a ligand, followed by a multi-objective genetic algorithm approach to modify torsion angles around rotatable bonds, stereochemistry of double bonds, chiral centres, and ring conformations. Some other tools that were developed for ligand preparation include Prepflow [144], VSPrep [145], Gypsum-DL [146], Frog2 [147] and UNICON [148].

#### 2.2.3. Protein Flexibility

Protein flexibility is essential for their biological function and subtle changes, such as side-chain rearrangements, can alter the size and shape of the binding site and thus bias docking results [149]. Methods to handle protein flexibility can be divided into four groups: soft docking [150,151], side-chain flexibility [152], molecular relaxation [153], and protein ensemble docking [154,155]. Soft docking allows small degrees of overlap between the protein and the ligand by softening the interatomic vdW interactions in docking calculations [151]. These are the simplest methods and are computationally efficient, but they can only account for small changes. Side-chain flexibility allows the sampling of side-chain conformations by varying their essential torsional degrees of freedom, while the protein backbones are kept fixed [156]. The molecular relaxation method involves both protein backbone flexibility and side-chain conformational changes; it first uses rigid-protein docking to place the ligand into the binding site then relaxes the protein backbone and the nearby side-chain atoms, usually employing methods, such as MC and MD [157,158,159]. Protein ensemble docking methods dock the ligand on a set of rigid protein structures, with different conformations which represent a flexible receptor. The docking results for each conformation are then re-analysed [160].

Most contemporary docking approaches treat proteins with partial or complete flexibility. For instance, Schrödinger offers a range of docking methodologies with different treatment of protein flexibility. Glide [94,95], with standard precision (SP) and extra precision (XP) is a docking strategy, which allows conformational flexibility for the ligands but treats the receptor as a rigid entity. It softens the active site via vdW scaling (soft docking) with the option of rotamer configuration sampling. Meanwhile, a superior method, Induced Fit Docking, uses Glide for docking to account for ligand flexibility, and Prime [161,162] for side-chain optimisation to account for receptor flexibility [163]. The ligand is docked into the receptor using Glide with vdW scaling and flexible side-chains are temporarily mutated to alanine to reduce steric clashes and the blocking of the binding site. Once the docking poses are generated, the mutated residues are restored to their original residues and Prime (a program for protein structure predictions) [161,162] is used to predict and reorient the side-chains with each ligand pose. The ligand–receptor complex is then minimised to afford a low-energy protein conformation, which is used for ligand resampling with Glide.

Water molecules have a crucial role in biological systems and interactions, such as stabilising protein–ligand complex, biomolecular recognition and participating in H-bond networks. Water molecules can participate in ligand–protein interactions by acting as bridging waters, and their displacement from the binding site upon ligand binding can also contribute to binding affinity, playing a significant role in the thermodynamics of protein-ligand binding [164]. The retention or removal of water molecules during virtual screening can have a direct impact on the size, shape and chemical properties of the binding site, which can influence binding geometries and affinity calculations.

Due to the ability of a water molecule to act as both an H-bond donor and acceptor, as well as its highly mobile nature, predicting the location and contribution of water molecules in protein–ligand binding is a challenging task. Crystal structures or cryo-EM structures of proteins can sometimes capture the placement of water molecules in the protein matrix, but the information is not always accurate due to the low resolution of the structural data, and the sample preparation conditions do not reflect the biological environment [165,166,167,168].

Many approaches were developed to simulate and predict the behaviour of water molecules. Implicit models, also known as continuum models, treat water molecules as a uniform and continuous medium. The free energy of solvation is traditionally estimated based on three parameters, the free energy required to form the solute cavity, vdW interactions and electrostatic interactions between solute and solvent. This method is less computationally demanding but neglects details at the solute–solvent interface [167,168]. Explicit models are computationally more expensive, but the molecular details of each water molecule are considered. Water molecules are normally described using a three-, four-, or five-point model.

In protein–ligand docking, water can be treated explicitly or in an approach involving a combination of implicit and explicit (hybrid), and they can be separated into four categories: (1) Empirical and knowledge-based methods (e.g., Consolv [169] and WaterScore [170]), (2) statistical and molecular mechanics methods (e.g., GRID [171,172], 3D-RISM [173,174], SZMAP [175]), (3) MD simulation methods (e.g., WaterMap [176], GIST [177], SPAM [116]) and, lastly, (4) Monte Carlo simulation methods (e.g., JAWS [178]).

#### 2.2.4. Scoring Functions

After searching for all possible binding modes, a scoring function is used to evaluate the quality of the docking poses. Scoring functions determine the binding mode and estimate binding affinity, which assists in identifying and ranking potential drug candidates. There are three main categories of scoring functions: force field-based, empirical-based, and knowledge-based methods.

Force field-based scoring functions generally use standard force field parameters taken from force fields, such as AMBER [179], which consider both the intramolecular energy of the ligand and the intermolecular energy of the protein–ligand complex [180]. The Δ*G* estimated using this scoring function is the sum of these energies, which is generally composed of vdW and electrostatic energy terms. An example of program that uses this method is DOCK, which utilises the following equation: [181,182]

(1) $$\Delta G=\underset{i}{\sum }\underset{j}{\sum }\left(\frac{A_{ij}}{r_{ij}^{12}}\;-\;\frac{B_{ij}}{r_{ij}^{6}}+\frac{q_{i}q_{j}}{\varepsilon \left(r_{ij}\right)r_{ij}}\right)$$

where $r_{ij}$ is the distance between protein atom i and ligand atom j, $A_{ij}$ and $B_{ij}$ are vdW components (repulsive and attractive vdW), $q_{i}$ and *q_j_* are atomic charges and $\varepsilon \left(r_{ij}\right)$ is the distance-dependent dielectric constant.

Empirical-based functions estimate binding affinity based upon a set of weighted energy terms that are described in the following equation:

(2) $$\Delta G=\underset{i}{\sum }W_{i}\;\cdot \;\Delta G_{i}$$

The energy terms ($\Delta G_{i}$) represents energy terms such as vdW energy, electrostatic energy, hydrogen (H) bond interactions, desolvation, entropy, hydrophobicity, etc., whereas the weighting factors ($W_{i}$) are determined via regression analysis by fitting the binding affinity data of a training set of protein–ligand complex with known 3D structures [94]. The first empirical scoring function (SCORE) was developed by Böhm in 1994 [183] based upon a dataset of 45 protein–ligand complexes, and the scoring function considers four energy terms: hydrogen bonds, ionic interactions, the lipophilic protein–ligand contact surface and the number of rotatable bonds in the ligand. Over time, the empirical scoring function has evolved by expanding the data set and considering more energy terms. For example, ChemScore, developed by Eldridge et al. [184], also considers metal atoms contribution and Glide XP score includes terms to account for desolvation effects [94].

In knowledge-based functions, structural information is extracted from experimentally determined structures of protein–ligand complexes from databases, such as the PDB [30] and Cambridge Structural Database (CSD) [185,186]. Boltzmann law is employed to transform the protein–ligand atom pair preferences into distance-dependent pairwise potentials, and the favourability of the binding modes of atom pairs is related to the frequency observed in known protein–ligand structures [187,188]. The potentials are calculated using the following equation:

(3) $$w\left(r\right)=-{\;K}_{B}T\ln\;\left[g\left(r\right)\right],\;g\left(r\right)=\rho \left(r\right)/\rho *\left(r\right)$$

where w(r) is the pairwise potential between protein and ligand, $K_{B}$ is the Boltzmann constant, T is the absolute temperature of the system, ρ(r) is the number density of the protein–ligand atom pair at distance r, and ρ∗(r) is the pair density in a reference state where the interatomic interactions are zero. 

**Table 1:** List of common docking programs.

Program	Ligand Flexibility	Receptor Flexibility	Scoring Functions	Examples of Application
Glide (HTVS, SP and XP) [94,95,189]	Exhaustive ligand conformation search	Soft docking	Empirical	Discovery of novel fibroblast growth factor receptor 1 kinase inhibitors [190] and CDK5 inhibitors [191]
GOLD [93]	Genetic algorithm	Soft docking Ensemble docking Side-chain flexibility	Goldscore (empirical) Chemscore (empirical) ChemPLP (empirical) ASP (knowledge based)	Design of non-peptide MDM2 inhibitors [192]
Autodock 4 [193]	Genetic Algorithm Simulated Annealing Local Search Lamarckian Genetic Algorithm	Side-chain flexibility	Semi-empirical free energy force field	Discovery of reversible NEDD8 activating enzyme inhibitor [194]
DOCK 6 [195]	Incremental construction algorithm	Rigid	Force field	Design and development of potent and selective dual BRD4/PLK1 Inhibitors [196]
Internal Coordinates Mechanics (ICM) [197]	Stochastic search (MC)	Side-chain flexibility (rotamer libraries)	Force field	Discovery of novel retinoic acid receptor agonist [198] and enoyl-acyl carrier protein reductase inhibitors in *Plasmodium falciparum* [199]
Surflex [200,201]	Incremental construction algorithm	Ensemble docking	Empirical	Discovery of novel inhibitors of *Leishmania donovani* γ-glutamylcysteine synthetase [202]
MOE [99,203,204,205]	Systematic (exhaustive) Stochastic High throughput Conformational Import (incremental construction + stochastic) [99]	Rigid	ASE (empirical) Affinity dG (empirical) Alpha HB (empirical) GBVI/WSA (force field)	Identification of novel monoamine oxidase B inhibitors [206] and Chk1 inhibitors [207]
FlexX [208,209]	Incremental construction algorithm	Rigid	Empirical	Identification of PKB inhibitors [210] and phosphodiesterase 4 inhibitors [211]
FRED [212,213]	Systematic (exhaustive) search, precomputed using Omega (using torsion and ring libraries) [138]	Rigid	Chemgauss 3 (empirical) Chemgauss 4 (empirical)	Discovery of selective butyrylcholinesterase inhibitors [214]

Abbreviations: ASP: Astex Statistical Potential; BRD4: Bromodomain 4; CDK5: Cyclin dependent kinase 5; ChemPLP: Piecewise Linear Potential; HTVS: high throughput virtual screening; MDM2: Mouse double minute 2 homolog; PKB: Protein kinase B; PLK1: Polo-like Kinase 1.

## 3. Ligand-Based Drug Design

When there is limited structural knowledge on the target protein, biological and chemical information is drawn from known active ligands to identify key features that are responsible for biological activity and this information can be used for ligand-based drug design (LBDD). Common LBDD methods include similarity searches, scaffold hopping, quantitative structure–activity relationship (QSAR) and pharmacophore models. Although CADD approaches are generally classified as structure-based and ligand-based approaches, it should be noted that virtual screening strategies often integrate and combine the two to improve the success rate in hit identification [215].

### 3.1. Similarity Search

The underlying hypothesis of molecular similarity is that molecules with similar molecular structures have similar physical properties and biological activities. Two key components in similarity analysis are structural representations and quantitative measurements of similarity between the two structural representations.

Different molecular fingerprints can be used to represent the chemical properties of a molecule, and similarity measurements can rely on the use of 1D, 2D and 3D descriptors. This involves dividing the molecule into a sequence of bits; so, the common bits between molecules can be compared to assess similarity. Some common molecular fingerprints include structural keys, topological fingerprints, circular fingerprints and pharmacophore fingerprints [216]. Structural key fingerprints, such as the MACCS fingerprint [217] and TGD fingerprint [218], search for the presence of structures/features of the molecules based on a pre-defined list of structural keys. This method is most useful when the molecules contain a lot of structural keys. Topological fingerprints (e.g., Daylight fingerprint) [219] analyse the fragments of the molecule following a connectivity path (usually linear) up to a certain number. The algorithm generates a pattern for each atom in the molecule, then a pattern for each atom and its nearest neighbours and connecting bonds, followed by a pattern that represents each group of atoms and bonds connected by paths up to two bonds long, and the process continues with longer bond paths. Circular fingerprints, such as Molprint2D [220] and extended-connectivity fingerprints (ECFP) [221], look at the environment of each atom in the molecules up to a certain radius. Every heavy atom of a molecule is sequentially used as a starting point and is assigned an atom type. This is followed by the assignment of atom types to neighbouring atoms of the central heavy atoms (first layer). This process is repeated with each distance/layer from the central heavy atom and the number of atoms with each given atom type are recorded to calculate descriptor values [222]. In addition to the common molecular fingerprints mentioned that are mostly used to describe synthetic compounds, the Natural Compound Molecular Fingerprint (NC-MFP) was developed by Seo et al. to better represent natural products [223].

There are different metrics that can be used to assess and quantify the similarity between two molecules (A and B). Most metrics have the range from 0 (completely dissimilar) to 1 (identical). Some of the common metrics are listed below:

Tanimoto coefficient (range: 0–1): [224]

(4) $$S=\frac{c}{a+b-c}$$

Dice index (range: 0–1): [225]

(5) $$S=\frac{2c}{\left(a+b\right)}$$

Cosine coefficient (range: 0–1): [226]

(6) $$S=\frac{c}{\sqrt{ab}}$$

Euclidean distance (range: 0–1): [226]

(7) $$D=\sqrt{a+b-2c}$$

where a is the number of bits present in molecule A, b is the number of bits present in molecule B and c is the number of bits present in both molecule A and B. S denotes similarities and D denotes distances where $S=\frac{1}{1+D}$. The cut-off values for the similarity metrics depend on both the fingerprints and metrics used and hence cannot be compared directly. For example, WebCSD, the online portal to CSD, offers both the Tanimoto coefficient and the Dice index for similarity search and the default cut-off values were set as 0.7 and 0.975, respectively [227].

**Figure 7:**
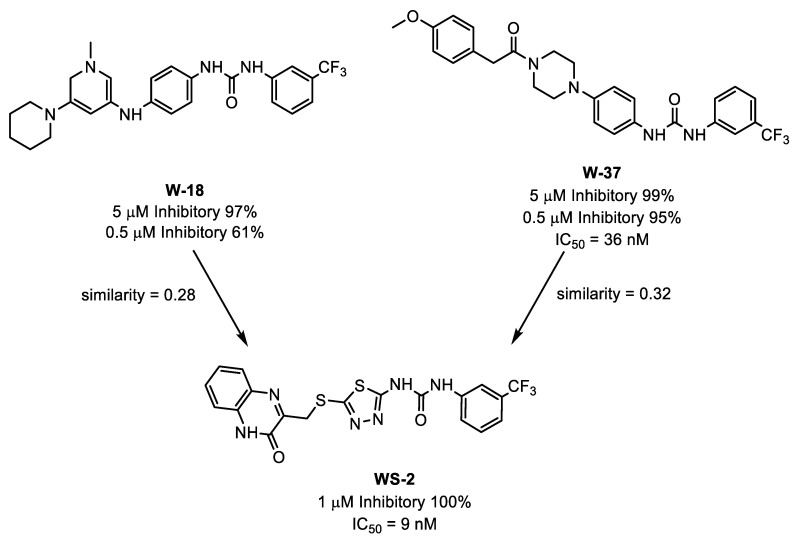
Discovery of the CDK8 inhibitor WS-2 from W-18 and W-37 using similarity search [228].

Wang and co-workers used a combination of docking-based and 2D similarity search techniques to identify novel CDK8 inhibitors [228]. A small molecule library was first subjected to molecular docking against multiple crystal structures of CDK8 to account for the protein conformation change. Of the 50 candidates selected from the docking study, 7 showed more than 30% inhibition against CDK8 based on *in vitro* binding competition assay. Similarity search using Discovery Studio [229] was performed on W-18 and W-37, two of the most potent candidates, to find similar structures with high CDK8 inhibitory effects. Using the Tanimoto coefficient to calculate the similarities of molecules based on the ECFP_6 fingerprints, WS-2 which shares 0.28 and 0.32 similarity with W-18 and W-37, respectively, was identified and it is significantly more potent than both of the parent molecules (Figure 7).

### 3.2. Quantitative Structure-Activity Relationship (QSAR)

A QSAR model is a computational or mathematical model that derives correlation between the calculated molecular properties of a group of compounds and their experimentally determined activity. QSAR methodology was first proposed by Hansch and Fujita in 1964 who published a method for the correlation of biological activity and chemical structure [230], and QSAR methodology has evolved a lot since then. 1D- and 2D-QSAR models are classified as ‘classical’ QSAR methodologies, where 1D-QSAR correlates biological activity with molecular properties, such as pKa and logP [231], and 2D-QSAR correlates biological activity with the structure of the ligands on a 2D basis and considers descriptors, such as topological and constitutional descriptors [230,232]. Topological descriptors are based on the connectivity of atoms in the molecule, including molecular size, shape, branching, heteroatoms and multiple bonds but with no information on the 3D spatial arrangement of the atoms [181]. Constitutional descriptors simply describe the molecular composition of a molecule, such as molecular weight, number of atoms and bonds, types of atoms, and ring counts.

3D-QSAR takes into account the 3D spatial representation of molecules, such as different conformations and stereo-isomerisation. Two of the most popular 3D-QSAR methodologies are the Comparative Molecular Field Analysis (CoMFA) proposed by Cramer et al. [233] and the Comparative Molecular Similarity Indices Analysis (CoMSIA) proposed by Klebe et al., a modified version of CoMFA [234]. The primary goal of 3D-QSAR is to establish a relationship between biological activity and spatial properties of the ligands, therefore data quality and structural diversity are particularly important to construct a good quality 3D-QSAR model. 3D-QSAR is often used for lead optimisation and biological activity prediction for novel compounds as it can quantitatively correlate modifications in 3D chemical structures and the respective changes in biological effects.

For example, the 3D-QSAR method was applied in the structure–activity relationship (SAR) analysis of maslinic acid analogues and the identification of its anti-cancer target. Maslinic acid analogues are known to be anti-cancer compounds but there was no structural information about its molecular target. A common pharmacophore model on five analogues was first constructed, then field points-based descriptors were used to build a 3D-QSAR model after aligning 74 analogues to the pharmacophore model. A field point-based similarity search on maslinic acid was performed on the ZINC database, followed by screening through the 3D-QSAR model for bioactivity prediction and SAR field point’s compliance. Additional filters (Lipinski’s rule of five, absorption, distribution, metabolism, and excretion (ADME) and synthetic accessibility) were also applied and eventually 39 compounds were listed. The compounds were docked against a series of potential cellular targets of maslinic aid analogues (predicted by STITCH) [235] and identified NR3C1 as a major anti-cancer target of maslinic acid analogue as well as compound P-902 as a potential lead compound [236].

### 3.3. Pharmacophores

**Figure 8:**
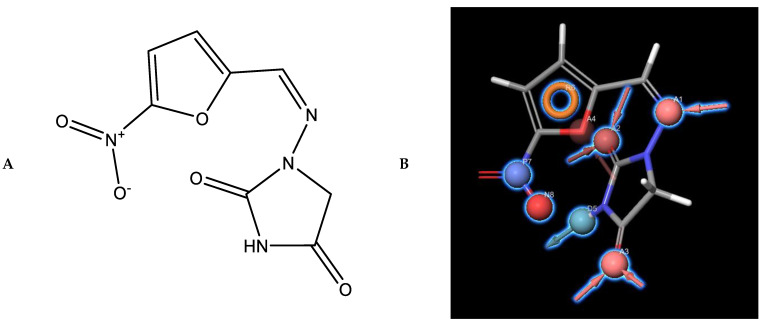
(**A**) Chemical structure of nitrofurantoin. (**B**) Nitrofurantoin superimposed with pharmacophore features. Light red sphere: H-bond acceptor; light blue sphere: H-bond donor; red sphere: negative ionic; blue sphere: positive ionic; orange torus: aromatic ring.

It is widely believed that Paul Ehrlich came up with the concept of pharmacophore: a molecular framework that carries (*phoros*) the essential features responsible for a drug’s (*pharmacon*) biological activity in the early 1900s [237,238]. However, some consider the concept of modern pharmacophore was in fact proposed by Schueler in 1960 [239], which was then extended by Beckett and co-workers who introduced the first pharmacophore model with identified distance ranges in 1963 [240] and Kier who proposed the first computed pharmacophore model in 1967 [241]. Nowadays, pharmacophore models are extractions of electronic and steric features from ligands in a 3D spatial arrangement that is relevant for interactions to the target protein and the relative biological responses. The features are purely abstract concepts and do not represent chemical functional groups or a typical structural skeleton [242]. The six classical pharmacophore features classified are H-bond donors, H-bond acceptors, negative ionic, positive ionic, hydrophobic regions, and aromatic regions (Figure 8). On top of that, less common features can also better characterise the chemical functionalities, such various metal binding locations are supported by LigandScout [243,244,245,246]. Constraints and restrictions can also be applied by introducing excluded volumes to the model to prevent ligands from occupying certain spaces (ligand-inaccessible) [247]. Pharmacophores can be divided into two sub-categories: ligand-based and structure-based pharmacophores.

Ligand-based pharmacophores are based on the chemical structures of ligands when there is little structural information about the target protein is available. To construct a ligand-based pharmacophore, the conformational space of flexible active molecules (through conformational sampling) is covered because the molecules should be in their bioactive conformations. The molecules are then aligned, and common features are extracted to generate a pharmacophore model. Alignment techniques are divided into point-based and property-based approaches [248]. In point-based approach, atoms, fragments, or chemical feature point distances are minimised, and pairs of points are superimposed by minimising distances. Some examples of programs that use point-based alignment include HipHop [249], Phase [250,251] and Galahad [252]. In contrast, property-based approaches (e.g., MOE [99]) generate alignments based on molecular field descriptors, such as electron density, electrostatic potential, molecular shape and volume, etc. [248,253].

A study conducted by Rampogu et al. developed a ligand-based pharmacophore model for the screening of natural compounds against HER2 kinase domain. A total of 82 compounds with various levels of activity were chosen from the literature where 32 of them were used to construct a pharmacophore model using Discovery Studio [229]. The rest of the compounds, together with decoy set, were employed to validate the model. A total of 197 201 compounds from the Universal Natural Products Database were first filtered for ADME and Lipinski’s Rule of Five to identify compounds with drug-like properties, followed by screening against the pharmacophore model. The resulting compounds were subjected to molecular docking and MD simulations and eventually identified two potential leads against HER2 breast cancers [254].

One of the biggest drawbacks in ligand-based pharmacophore modelling is the selection of training set ligands. Searching for active ligands to form a training set from the literature could be a difficult task as biological assays were conducted under different experimental conditions. Performing biological assays, such as enzyme kinetic assays, under consistent conditions can be useful to investigate relative biological activities of the ligands, and hence the direct ligand–protein interactions. Although highly different pharmacophore models can increase diversity and cover wider chemical space, training set ligands with larger structural differences might require other experimental validation (e.g., X-ray crystal structure) to confirm they share the same binding site. Nevertheless, the real problem of ligand-based pharmacophore modelling lies in defining if a ligand is active or inactive, particularly in the case of defining qualitative pharmacophores. The diversity of the dataset could hugely affect the pharmacophore model generated, including feature types, locations and excluded volumes [255].

Compared to ligand-based pharmacophores, structure-based approaches are less likely to be biased by the chemical structures of existing active compounds and thus yield more diverse molecules. Structure-based pharmacophore models are constructed from either a protein–ligand complex or from the 3D structure of the receptor alone (receptor-based). The protein–ligand complex approach evaluates the key interactions between the ligand and the binding site and then transforms this information into a pharmacophore model [256]. For cases where the structural information of the ligand is lacking, the receptor-based approach can be applied. Pharmacophore hypotheses can be derived from protein structures using two methods: geometric constraints [257] and binding site analysis using virtual probe atoms [258].

MurG is one of the enzymes involved in the biosynthesis of the peptidoglycan layer in *Mycobacterium tuberculosis* and inhibition of MurG could be useful for the treatment of tuberculosis. Saxena et al. built a pharmacophore model based on the protein–ligand interactions of the homology model of *Mycobacterium tuberculosis* MurG due to the lack of available crystal structures. The pharmacophore model, along with molecular docking and MD simulations, was used and identified three lead compounds that were potential *Mycobacterium tuberculosis* MurG inhibitors [259].

#### 3.3.1. Pharmacophore Validation

Before employing the pharmacophore model for virtual screening, it is essential to validate the model to evaluate the predictivity of the model. Decoys databases such as DUD-E [260], MUV [261] and DEKOIS [262] are often used to test the model’s ability to differentiate active and inactive compounds. Multiple refinements are frequently performed to result a better model after testing with different metrics. Examples of some of the commonly used metrics are listed below:

Yield of actives (Ya) shows the retrieved true positive compounds (Ha) in relation to the number of hits retrieved (Ht) [263].

(8) $$Ya=\frac{Ha}{Ht}$$

Sensitivity (Se) is the ratio *Ha* to all the actives compounds (A) in the database. The closer the number is to 1, the higher number of active compounds returned from the search. It gives an insight into the ability of the model to select truly active compounds [264].

(9) $$Se=\frac{Ha}{A}$$

Specificity (Sp) is the ratio of rejected true negatives (TN) to all the–inactive compounds (D−A), where D is the number of entries in the database). When Sp = 1, all the inactive compounds have been correctly rejected. Specificity tells us the ability of the model to discard inactive compounds [264].

(10) $$Sp=1-\frac{Ht-Ha}{D-A}$$

Enrichment factor (EF) measures Ya proportional to the ratio of A in the whole database [264].

(11) $$EF=\frac{Ya}{A/D}=\frac{Ha/Ht}{A/D}$$

The Goodness of Hit list (GH score) is a combination of sensitivity, specificity, and yield of actives of different weightings. It considers both true actives ratio and true inactives ratio, which makes it a very powerful tool [265]. The GH score ranges from 0 (null model) to 1 (ideal model), a model with a GH score > 0.6 is generally expected to be reliable [266].

(12) $$GH\;score=\left(\frac{3}{4}\cdot Ya+\frac{1}{4}\cdot Se\right)\cdot Sp=\left(\frac{Ha\left(3A+Ht\right)}{4HtA}\right)\left(1-\frac{Ht-Ha}{D-A}\right)$$

**Figure 9:**
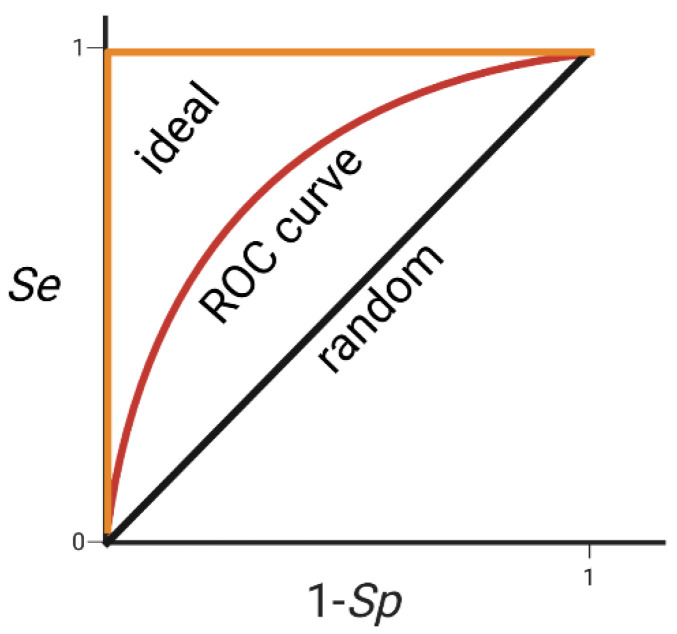
An example of Receiver operative characteristic (ROC) curve. Black: random classifier; orange: ideal curve; red: ROC curve.

The Receiver operative characteristic (ROC) curve displays the increase of false positives that results with increased true positives. On the *Y*-axis the true-positive rate (Se) is represented, and on the *X*-axis the false-positive rate (1 −  Sp) is represented. The area under the curve (AUC) is normally used to measure the performance of the model. The greater the AUC (ideal value is 1), the better is the model. An AUC of 0.5 indicates a random database search and thus a poor model (Figure 9) [267].

#### 3.3.2. Pharmacophore Screening

Once the model is validated, databases of small molecules are screened against the pharmacophore model (query) and molecules that match with the features in the model will be extracted and identified as hit compounds. Other than ligand alignment, conformational flexibility is the other big challenge that is encountered in pharmacophore-based virtual screening. In general, conformations of ligands can either be pre-enumerated before the screening process, or conformation search is performed on-the-fly in the pharmacophore fitting process [268]. Pre-enumeration is less computationally expensive but requires bigger storage space, whereas conformation search on-the-fly is time consuming and requires more computer power. Some common pharmacophore screening programs include Catalyst [249], Phase [250,251], LigandScout [269], PharmID [270].

**Figure 10:**
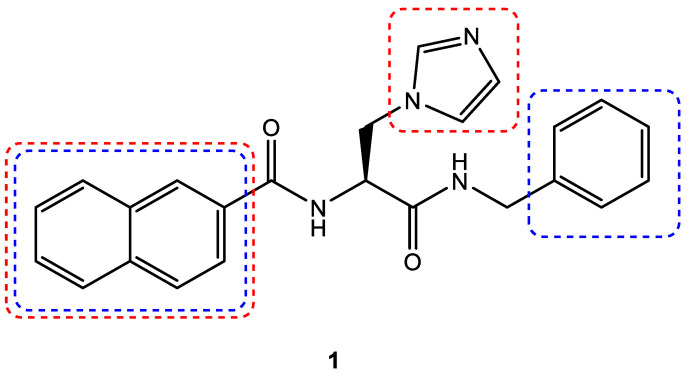
Chemical structure of **1**. Naphthyl, phenyl and imidazole fragments that match with pharmacophore features of the squalene cyclooxygenase model (blue) and CYP51 model (red), respectively, were connected to give **1** with dual-target inhibition [271].

Recently, Dong and co-workers discovered an anti-fungal inhibitor that can inhibit both squalene cyclooxygenase and CYP51 using pharmacophore modelling. First, a ligand-based common feature pharmacophore model was generated for squalene cyclooxygenase based on seven known inhibitors with diverse scaffolds. Next, a structure-based pharmacophore model for CYP51 was generated from the crystal structure of CYP51 and its interaction with the co-crystalised ligand itraconazole (PBD ID: 5V5Z). Fragments were selected and superimposed onto the pharmacophore features of each of the model and one was constructed by linking different fragments from each of the two models generated, and it was found to inhibit both enzymes simultaneously (Figure 10) [271].

Unlike molecular docking, which has well developed scoring functions to estimate binding affinity, pharmacophore screening only assesses how well the ligand matches with the pharmacophore model and that is commonly done by calculating RMSD. Some programs also implement penalties and weightings based on different features, such as the fitness score from phase is based on RMSD, vector terms and volume terms [250,251]. Nevertheless, visual inspection and other criteria such as ADME [272] and pan-assay interference compounds (PAINS) [273] are often required to filter inappropriate hits.

### 3.4. Scaffold Hopping

Scaffold hopping (lead hopping) is a technique that identifies iso-functional molecular structures with significantly different molecular backbones [274]. The process normally starts with a known active compound, and by replacing with different “cores” (scaffold hopping), a structurally novel compound with similar biological activity is created. The search for alternative cores can be carried out using other LBDD methodologies, such as pharmacophore searching, shape screening and similarity searching using 2D or 3D fingerprints.

In scaffold hopping, the degree of change of the new molecule compared to the original parent molecule ranges from minor changes, such as heterocycle replacement to extensive modifications like topology-based hopping which creates molecules with significantly different scaffold. Sun et al. classified scaffold hopping into four categories based on the degree of modification [275]. Heterocycle replacement is defined as 1° hopping. Even though there are limited changes in properties of the molecule, it often accompanies a high success rate and an increase in binding affinity to the target protein. 2° hopping involves ring opening and closure which could be useful for adjusting molecular flexibility. 3° hopping are a substitution of pseudopeptides or peptidomimetics that replaces the peptide backbone of the parent molecule with nonpeptic moiety. 4° hopping is topology-based and produces molecules with new chemical backbones to the parent drug, which could present novel properties.

**Figure 11:**
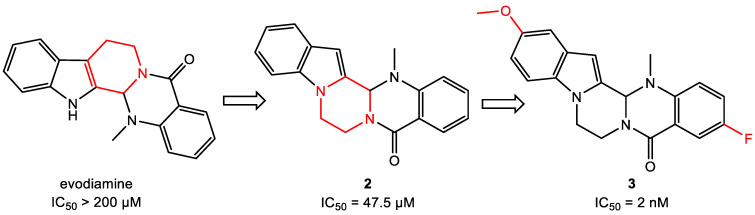
Design and optimisation of indolopyrazinoquinazolinone derivatives from evodiamine using scaffold hopping [278].

Scaffold hopping is particularly useful in optimising known ligands to improve their efficacy and ADME profile [276]. Blaquiere et al. discovered novel NF-κB inducing kinase (NIK) inhibitors with improved selectivity and pharmacokinetic properties using the scaffold hopping method. By replacing the oxepin ring in their previously discovered benzoxepine class NIK inhibitors with different cores, novel molecules with reduced nonoxidative metabolism (glutathione conjugation and amide hydrolysis) and thus reduced *in vitro* clearance were identified [277]. Scaffold hopping is also an effective strategy to optimise natural products with insufficient levels of activity and high structural complexity to increase their potency and synthetic accessibility. By changing the connectivity of the piperidine ring of natural product evodiamine, Wang and co-workers identified a novel indolopyrazinoquinazolinone scaffold **2** with anti-tumour properties, bringing the IC_50_ value from over 200 µM to 47.5 µM when tested against HCT116 cells. Further structural optimisation resulted in a molecule **3** with an IC_50_ value of 2 nM (Figure 11) [278].

## 4. *De Novo* and Fragment-Based Drug Design

*De novo* drug design allows the generation of novel molecules with new scaffolds, especially when majority of small molecule libraries have been exhausted for virtual screening. Before performing *de novo* drug design, the primary target constraints must be determined first. In SBDD where the structure of the receptor is known, molecular shapes, sub-molecular physical and chemical properties that are important for binding to the active site are extracted to derive shape constraints and interaction sites (normally divided into H-bonds, electrostatic and hydrophobic interactions). In LBDD, pharmacophore features can be used directly in a similarity design method or treated as interaction sites and generate a pseudo-receptor model [25].

Building blocks used for the generation of molecules can either be atoms or organic fragments. Early programs mainly used atom-based approach that is more likely to encounter issues with synthetic accessibility, but the molecules generated would be more diverse as all chemical space can be sampled. Newer programs use a fragment-based approach that is generally more synthetically feasible but the resulting molecules are relatively less diverse [279]. Furthermore, using fragments obtained by cleaving drug molecules had shown to generate ligands that are more likely to have drug-like properties [280]. Some examples of *de novo*/fragment-based drug design programs include LUDI [257], LigBuilder [281], ACFIS [282] and SEED [283].

Structure sampling can be carried out in various methods: linking, growing and lattice-based sampling. The linking approach links the building blocks that are positioned at the interaction sites with linker to form a complete molecule [257,284]. The growing approach starts off with one building block that is positioned at one of the interaction sites (starting point), then the structure grows from the starting point, trying to fit suitable interactions for the interaction sites as well as the regions of the receptor between interaction sites [285,286]. The lattice strategy places the binding pocket with lattice points and the ligands are formed from the lattice points that lie along the shortest path that connects the interaction points [287]. Once the molecules are generated, they can either be assessed with structure-based methods to predict the binding affinity, or with ligand-based methods where the molecules are compared to known active compounds. The ligands are then optimised until a promising drug candidate is produced.

**Figure 12:**
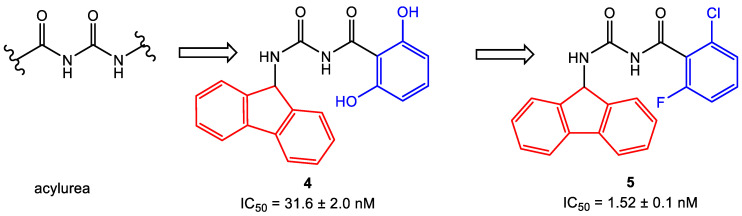
Discovery process of **4** and **5**.

Ni and co-workers discovered a new class of Cyclophilin A (CypA) inhibitors using *de novo* drug design approach with LigBuilder 2.0. Analysis of existing CypA inhibitors shows that potent inhibitors contain an amide fragment as a linker that forms H-bond interactions with residues between the two sub-binding pockets. Using an acylurea linker as the starting point, new molecules are generated by growing structures from both ends of the structures to occupy the two sub-binding pockets of CypA. Out of the top 98 molecules that were generated, a common scaffold **4** was identified. Compound **4** was found to be potent and was further optimised based on SAR information to give **5** that was 20 times more potent (Figure 12) [288].

## 5. Hierarchical Virtual Screening (HLVS)

Both structure-based and ligand-based virtual screening methods have their own strengths and weaknesses. Structure-based methods are dependent on the availability of protein structures and it could be computationally demanding and time consuming for methods, such as MD and flexible molecular docking. Docking-based methods also demonstrate varying performance depending on the nature of the target binding sites [289]. Ligand-based screening methods on the other hand, rely heavily on the knowledge and information of active ligands and as a result are more biased towards the chemical scaffolds of available active compounds and generate less diverse results. Unlike docking studies where there are well established scoring functions used to approximate binding affinity and to rank molecules, pharmacophore methods lack a reliable and general scoring system. There can also be a lot of variations in models generated in ligand-based approaches, for example a slight difference in ligand selection in the training set could generate a very different QSAR model.

**Figure 13:**
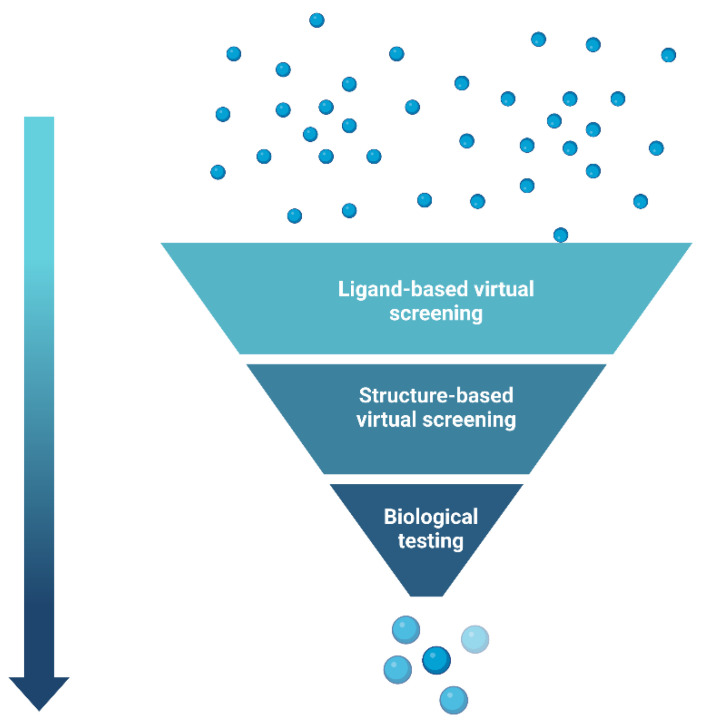
HLVS approach. A series of filters are sequentially applied to a database of small molecules to reduce the number of molecules to be taken to biological testing and extract lead compounds for further investigation and optimisation.

There are clear benefits to combine and integrate different approaches in CADD and the most common way is to use ligand- and structure-based methods in a sequential order, commonly known as hierarchical virtual screening (HLVS) (Figure 13). Generally, ligand-based filters are first applied because they are fast and less computationally expensive. Once the number of candidates is reduced, structure-based methods are applied to further filter inappropriate drug candidates before taking them for biological testing [290]. The hierarchical combination of pharmacophore modelling and molecular docking are the two most extensively employed methods in HLVS and there are numerous successful examples using this approach, such as the identification of matrix metalloproteinase 2 (MMP2) inhibitors by Di Pizio et al. [291] as well as the discovery of novel PKR-like endoplasmic reticulum kinase (PERK) inhibitors by Wang et al. [292].

## 6. Molecular Mechanical/Generalised Born Surface Area (MM-GBSA)

A more robust way to estimate the binding free energy of ligands to protein is to use the combined Molecular Mechanical/Generalised Born Surface Area (MM-GBSA) approach [293]. MM-GBSA is a force-field based method that computes the free energy of binding from the difference between the free energies of the protein, ligand, and the complex in solution. The free energy is calculated by using a combination of gas-phase molecular mechanics (MM) energy, electrostatic solvation energy (GB) and non-electrostatic contribution to solvation energy (SA). It provides a more accurate prediction because it can treat both the ligand and protein as flexible, allowing structural rearrangements required for the induced-fit pose. For the same reason, MM-GBSA is more computationally expensive compared to conventional docking studies, therefore they are generally implemented after a completed docking study to re-score selected ligands.

The total binding free energy ΔG_bind_ can be calculated using the following equation: [293]

(13) $$\Delta G_{\mathrm{bind}}=E_{\mathrm{complex}}-E_{\mathrm{ligand}}-{\;E}_{\mathrm{receptor}\;}$$

where $E_{\mathrm{complex}}$ is the energy of the optimised complex, $E_{\mathrm{ligand}}$ and $E_{\mathrm{receptor}\;}$ are the energy of the optimised free ligand and receptor, respectively. This equation can be further broken down into different components of the contributing energies:

(14) $$\Delta G_{\mathrm{bind}}=\Delta H\;-\;T\Delta S\;=\Delta E_{\mathrm{MM}}+\Delta G_{sol}-T\Delta S$$

In which

(15) $$\Delta E_{\mathrm{MM}}=\Delta E_{\mathrm{int}}+\Delta E_{\mathrm{ele}}+\Delta E_{\mathrm{vdW}}$$

(16) $$\Delta G_{sol}=\Delta G_{PB/GB}+\Delta G_{SA}$$

(17) $$\Delta G_{SA}=\;\gamma \cdot \mathrm{SASA}+b$$

where $\Delta E_{MM}$ is the changes in the gas-phase molecular mechanics (MM) energy, including changes in the internal energy ($\Delta E_{\mathrm{int}}$), electrostatic energy ($\Delta E_{\mathrm{ele}}$) and vdW energy ($\Delta E_{\mathrm{vdW}}$). $\Delta G_{sol}$ is the sum of electrostatic solvation energy with polar contribution ($\Delta G_{PB/GB}$) and non-polar contribution ($\Delta G_{SA}$) between the solute and continuum solvent. $\Delta G_{PB/GB}$ is calculated using either the Poisson-Boltzmann (PB) or generalised Born (GB) model. $\Delta G_{SA}$ is estimated using the solvent-accessible surface area (SASA), where γ is the surface tension constant and b is a correction constant. The change in conformational entropy (−TΔS) is calculated by normal-mode analysis [294,295,296].

## 7. Molecular Dynamics

Molecular dynamics (MD) is an *in silico* simulation method based on molecular mechanics (MM), to study the individual particle motions of model systems over time [297]. MD can provide insights into biomolecular processes, such as protein folding, conformational changes, ligand binding and disassociation by simulating the interactions between atoms and molecules at an atomic level [298,299,300,301]. In the context of drug design, simulating the responses of proteins to various perturbations, including mutation [302], phosphorylation [303], protonation [304] and ligand binding [305], can be observed in well-established models, making MD a powerful tool in understanding the mechanisms for pathogenic or therapeutical processes. Since initially being applied in macromolecules [306], the application of MD simulation has been extensively developed, both in algorithms and force field parameters. A variety of MD software packages are available such as Gromacs [307], AMBER [308], Lammps [309], NAMD [310], CHARMM [311] and Desmond [312]. These mainstream programs for MD simulation share similar functionalities and have achieved high performance by utilising the compute power and speed of graphics processing units (GPUs). MD has been gradually accepted and is now widely used in pharmaceutical science especially with recent breakthroughs in both structural biology techniques (leading to a larger number of experimentally obtained protein structures) and computational hardware. Currently, MD simulation is integrated into the crucial pilot stages of drug discovery [313]. Two major usages of MD in recent novel drug design are: (1) to provide dynamic structural insights of biomolecules and (2) to provide precise energetic information of receptor–ligand complexes, key information in lead identification and lead optimisation.

In this context, MD provides valuable time-dependent information on drug targets and their ligands [314]. MD simulations calculate the position and motion of each atom at each timestep. With accurately controlled simulation conditions, MD can capture the binding processes in action, which are difficult to observe experimentally, provide the details, such as the path in which ligand slides into the binding pocket [315], how the protein–ligand intermediate state forms and evolves [316], giving explanation of the binding mechanism at atomic resolution.

For the ligand binding processes, MD always works hand-in-hand with molecular docking [317]. As previously mentioned, the flexibility of a protein structure is a fundamental factor in both protein biological function and the shape of a binding pocket. However, the initial protein structure used in SBDD are usually the state of the protein acquired from experimental methods, such as X-ray crystal diffraction or cryo-EM [313]. In reality, different states of the protein exist and the protein dynamics profoundly affect the binding process. Docking into a single static structure would likely retrieve only one subset of promising ligands.

There are two main hypotheses of the ligand recognition: conformational selection and induced fit mechanism, which may coexist in most cases [318]. MD combined with ensemble docking is one solution to address receptor flexibility by conducting simulations to explore the conformational space and select representative conformations as a receptor ensemble into following dockings. This method is usually integrated in virtual screening workflows to enrich the structural diversity of lead candidates and possible rational binding poses [319]. Many successful practices of MD-based ensemble docking have been published. Li et al. conducted unrestrained MD simulations on estrogen-related receptor α (ERRα) to obtain structural ensembles for a virtual screening scheme which combines similarity search and ensemble docking. Seven novel scaffolds different from known agonists with remarkable activity were identified [320]. Recently, machine learning (ML) methodologies were also introduced to boost ensemble docking both on ensemble optimisation [321] and ligand score aggregation [322]. On the other hand, methods based on the induced fit mechanism, are also powered by MD simulation. Induced fit docking methods that aim to address the flexibility issue in ligand binding have been successfully utilised in many drug discovery projects [323,324]. However, the poses sampling step of classic IFD still leaves worries on robustness and accuracy. Thus, MD is introduced into the upgraded methodology called IFD-MD to overcome these challenges [325]. Compared to the traditional IFD protocol, in IFD-MD, short MD simulations are first applied in the rescoring procedure to equilibrate the trial binding models, then metadynamic simulations [326,327] are conducted to assess the local stability. This new method showed promising outcome both in efficiency and accuracy. Zhang et al. discovered dual agonist with nanomolar affinity on both orexin-1 and orexin-2 receptors and performed comprehensive computational modelling studies, including IFD-MD and conventional MD to explore the binding interactions [328].

Another important objective of MD is capturing conformational changes, particularly those related to important functional processes. As these biomolecular processes usually take place on a larger timescale than conventional MD can sample (within reasonable time and computational cost), several sophisticated MD schemes such as steered MD (sMD) [329], accelerated MD (aMD) [330], replica-exchange MD (REMD) [331] and coarse-grained MD [332] were developed to overcome the barrier [333]. In drug discovery, MD is widely used to explain biomolecular mechanisms, such as drug resistance caused by mutations [302,334,335,336]. Compared to time-consuming experimental method, which only gives static structural information, MD can rapidly provide detailed explanations of the interactions between the ligand and the receptor, including drug–protein or protein–protein interactions and not only structural and dynamical information but also energetic insights. Many studies verified the feasibility of using MD simulations in studying virus resistance mechanisms especially on recent COVID-19 topics. Liu et al. performed an all-atom MD simulation and free energy calculation to explain the resistance mechanisms of SARS-COV-2 variants Delta and Lambda to bamlanivimab [337].

The design of drugs targeting allosteric sites is another application of MD [305,338]. Allosteric binding sites are usually not as obvious as orthosteric sites from experimentally obtained structures, often due to their reliance on ligand binding and the induced conformational changes [333]. The formation of cryptic pockets is also considered being adjusted by both mechanisms [339]: conformational selection based on the flexibility of the cryptic pockets first and then stabilised by ligand as induced fit [340]. MD simulations have been shown to be of great use in identifying cryptic binding pockets and distinguishing allosteric and orthosteric sites [341,342]. Mixed solvents MD simulation, which uses small molecules/fragments with water as probe, have been successfully applied to detect and characterise allosteric sites [343,344]. Zuzic et al. used molecular dynamics simulations with benzene probes to detect the cryptic pockets in the SARS-CoV-2 spike glycoprotein and successfully identified a potentially druggable cryptic [345]. 

Protein misfolding is also an important topic that MD method is deeply involved. Unlike regular protein folding processes which have plenty of well-established solutions including homology modelling [58,59,60] and *ab initio* modelling [70,71,72,73], the high-resolution dynamic misfolding procedures of intrinsically disordered proteins (IDPs) are extremely difficult to be investigated in experiments for their heterogeneity [346,347]. Among all the cases, the pathological misfolding and aggregation of Alzheimer’s disease (AD) related amyloid-β (Aβ) peptide and tau protein are the most pressing areas for novel therapeutic agent development. Man et al. evaluated the effects of MM force fields on amyloid peptide assembly based on the experimental observation [348,349]. Liu et al. constructed the Markov state model based on the microsecond time scale MD simulation to explore the mechanism of VQIVYK (PHF6) peptide for tau protein aggregation [350].

**Figure 14:**
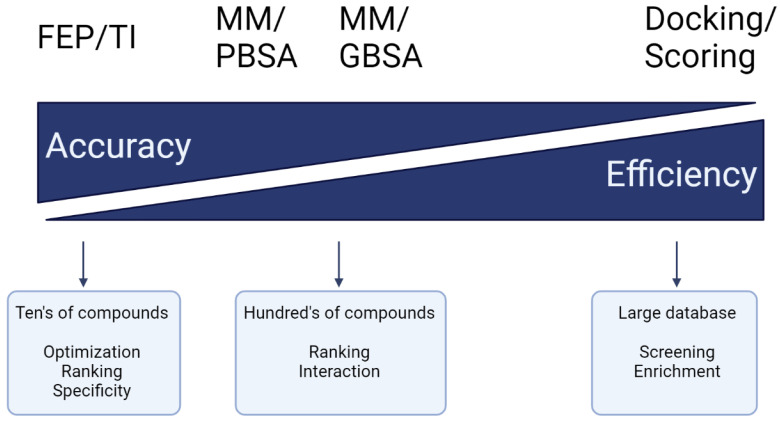
The distribution of the regular free energy calculation methods in accuracy/efficiency scale and their applications in drug discovery.

MD simulations can also contribute to lead candidates’ optimisation after the initial identification effort. Even though the structural information obtained by molecular docking provides insights into understanding the receptor-ligand interaction, the scoring functions suffered from their approximation in descripting desolvation, entropic penalties and conformational strains [351], leading to inaccurate energetic results in affinity prediction. The accurate evaluation of receptor–ligand interactions, along with the refinement of the binding complex structures, are needed, and they are becoming a standard protocol at the post-docking stage [352,353]. The purpose of MD optimisation is to fix clashes and stabilise and correct the binding complex, as well as to provide substantially accurate value of binding affinities by MD-based free energy calculations. Regular methods in this field consist of the alchemical approaches, such as thermodynamic integration (TI), free energy perturbation (FEP) [354,355,356,357] and endpoint approximation methods, such as molecular mechanics Poisson–Boltzmann (generalised Born) surface area (MM/PB(GB)SA) and linear interaction energy (LIE) (Figure 14) [295,358,359,360].

**Figure 15:**
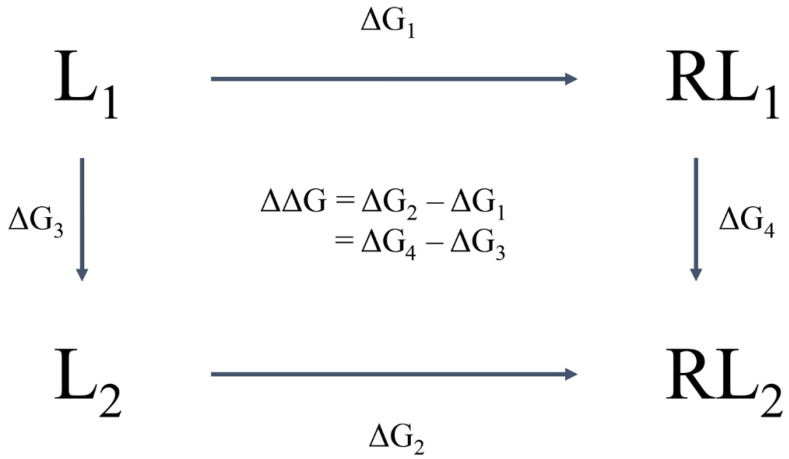
Thermodynamic cycle for relative binding free energy calculation. ΔG_1_ and ΔG_2_ are the binding energy of reference ligand L_1_ and modified ligand L_2_, respectively, ΔG_3_ is the free energy difference of two ligands in solution, ΔG_4_ is the free energy difference of two ligand–receptor complex in solution.

TI and FEP are theoretically rigorous methods with highly precise result. However, the sampling of the calculation requires a large amount of computing resources, and the system of computing is also limited because of the complicated simulations setup [361]. Usually, these methods are used to compare the free energy difference between two given systems with minor modifications, specifically, the lead optimisation process in drug design [362]. TI and FEP calculate the free energy difference between systems of similar chemical constitutions where the experimental data is not available. To accomplish the calculation, a thermodynamic cycle is introduced to connect the results from a series of TI calculations to experimental observables (Figure 15).

In this closed thermodynamic cycle, the free energy difference between two ligand binding ΔΔG can be calculated precisely as it is identical to ΔG_4_–ΔG_3_.

For two systems in TI approach, A and B, with potential energies $U_{A}$ and $U_{B}$, λ is introduced as the coupling parameter with value between 1 and 0, the new potential energy function is defined as

(18) $$U\left(\lambda \right)=U_{A}+\lambda \left(U_{B}-U_{A}\right)$$

In canonical ensemble, the partition function of the system is

(19) $$Q\left(N,V,T,\lambda \right)=\sum exp\;[-U\left(\lambda \right)/K_{B}T]$$

where $K_{B}$ is the Boltzmann constant, NVT means constant number (N), volume (V), and temperature (T).

The free energy of the system is defined as

(20) $$G_{\left(N,V,T,\lambda \right)}=-K_{B}TlnQ_{\left(N,V,T,\lambda \right)}$$

The free energy between A and B is calculated as

(21) $$\Delta G\left(A\to B\right)=\int_{0}^{1}d\lambda \frac{\partial F\left(\lambda \right)}{\partial \lambda }=\int_{0}^{1}d\lambda \frac{K_{B}T}{Q}\frac{\partial Q}{\partial \lambda }$$

(22) $$\Delta G\left(A\to B\right)=\int_{0}^{1}d\lambda \frac{K_{B}T}{Q}\sum \frac{1}{K_{B}T}\exp\;[-U\left(\lambda \right)/K_{B}T]\frac{\partial U\left(\lambda \right)}{\partial \lambda }$$

(23) $$\Delta G\left(A\to B\right)=\int_{0}^{1}d\lambda \langle {\frac{\partial F\left(\lambda \right)}{\partial \lambda }\rangle }_{\lambda }$$

For FEP method, a series of small perturbations, in our cases minor chemical structural modifications, are conducted to link the starting and ending state. The derivation is similar to TI

(24) $$G=-K_{B}TlnQ$$

(25) $$\Delta G\left(A\to B\right)=-K_{B}T\ln\left[\frac{Q_{B}}{Q_{A}}\right]$$

(26) $$\Delta G\left(A\to B\right)=-K_{B}Tln\left[\frac{\int e^{-U_{B}\left(\vec{q}\right)/K_{B}T}d\vec{q}}{Q_{A}}\right]$$

(27) $$\Delta G\left(A\to B\right)=-K_{B}T\;ln\langle e^{-\left(U_{B}\left(\vec{q}\right)-U_{A}\left(\vec{q}\right)\right)/K_{B}T}\rangle {}_{A}$$

where $\vec{q}$ is variable for coordinates and momentum.

Sophisticated solutions to drug discovery problems are provided by the application of TI and FEP. Nowadays most of the simulation packages support relative free energy simulations, including FEP plus within the Schrödinger suite [363], AMBER TI [364,365], CHARMM [366], Gromacs [367], Q open-source [368] MD package and so forth. Tang et al. utilised FEP to guide the discovery of novel D-amino acids oxidase inhibitors, with good consistency shown between bioassay results and the energy calculations [369]. Zou et al. developed a method for scaffold hopping transformations via alchemical free energy calculations, which broaden the usage of such approaches in lead modification and optimisation [370].

One issue that limits the utility of TI/FEP is the complicated set up for the system. Although the results of TI and FEP calculation are exact in theory, the accuracy is dependent on sampling/studying sufficient intermediate states that provide enough overlaps in each λ window. Different choices of λ are implemented, such as fixed value, slow growth and dynamic modified growth [371], improving the accuracy of the result, while also significantly increasing the computational cost. Even though GPU accelerated techniques have been applied in TI/FEP calculations [372], they are still not economically accessible for large datasets. Though MD software offered limited support to setup TI/FEP systems, much effort has been made to assist the preparation for TI/FEP calculations. Automated workflow tools, such as FESetup [373] for AMBER and Gromacs, PyAutoFEP [367] for Gromacs, FEPrepare [374] for NAMD, QligFEP [375] and QresFEP [376] for Q and many others [377,378,379] provide convenience for researchers to conduct alchemical free energy simulations in drug design.

MM/PB(GB)SA, as an endpoint method which depend on the sampling of the final states of the system, is a good trade-off for computational cost and accuracy in calculating the binding free energy [380]. The balanced performance makes it popular in broader utilities than the alchemical free energy methods [380,381,382].

**Figure 16:**
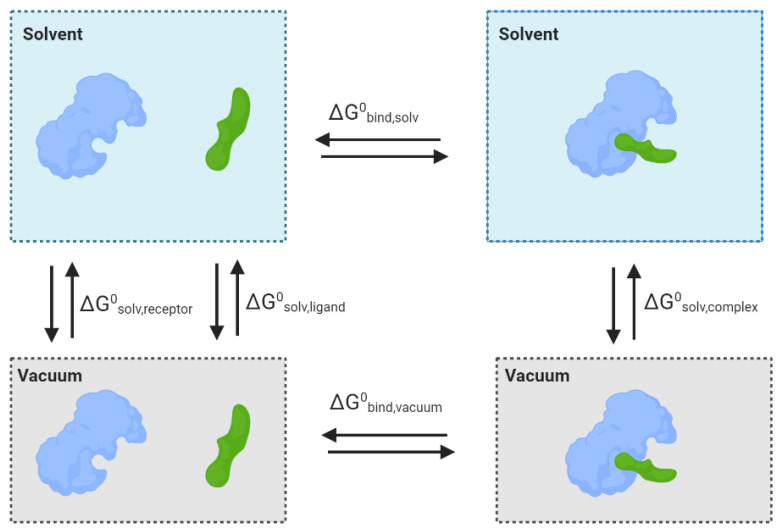
Thermodynamic cycle of binding free energy calculations for protein–ligand complex. ΔG^0^_bind,solv_ is the free energy of interest, solvation energy and binding energy in vacuum are directly calculated terms.

As the system is always in solvent, $\Delta G^{0}{}_{bind,solv}$ is almost impossible to calculate directly in explicit model because the majority of energetic contribution is made by the solvents instead of the complex, and the fluctuation of total energy is far beyond the binding energy [380]. Thus, the MM/PB(GB)SA also calculate through the thermodynamic cycle to avoid the problem (Figure 16).

(28) $$\Delta G^{0}{}_{bind,solv}=\Delta G^{0}{}_{solv,complex}-\;\left(\Delta G^{0}{}_{solv,ligand}-\Delta G^{0}{}_{solv,receptor}\right)+\Delta G^{0}{}_{bind,vacuum}$$

In which the total binding energy can be divided as solvation energy and gas phase MM energy ($\Delta G^{0}{}_{bind,vacuum}$).

For the MM energy

(29) $$\Delta G^{0}{}_{bind,vacuum}=\Delta G^{0}{}_{complex,vacuum}-\;\left(\Delta G^{0}{}_{receptor,vacuum}+\Delta G^{0}{}_{ligand,vacuum}\right)$$

(30) $$=\Delta H^{0}-T\Delta S^{0}$$

(31) $$=\left(\Delta E^{0}{}_{int}+\Delta E^{0}{}_{vdW}+\Delta E^{0}{}_{ele}\right)-T\Delta S^{0}$$

where $\Delta H^{0}$ is the enthalpy changes in the gas-phase molecular mechanics (MM) energy which is calculated statistically based on the trajectories produced by MD, $\Delta E^{0}{}_{int}$ stands for the internal energy including the bond, angle and dihedral, $\Delta E^{0}{}_{vdW}$ for vdW energy and $\Delta E^{0}{}_{ele}$ for electrostatic energy.$\;T\Delta S^{0}$ is the contribution of entropy, which can be obtained by normal mode analysis, quasi-harmonic analysis or quasi-Gaussian approach.

As for the solvation energy, which consists of the electrostatic, vdW and cavity effects, can be represented as nonpolar and polar terms which is calculated in a different manner.

(32) $$\Delta G^{0}{}_{solv}=\Delta G^{0}{}_{solv,vdw}+\Delta G^{0}{}_{solv,cav}+\Delta G^{0}{}_{solv,ele\;}=\Delta G^{0}{}_{solv,nonpolar}+\Delta G^{0}{}_{solv,polar}$$

In which the nonpolar energy $\Delta G^{0}{}_{solv,nonpolar}$ is easy to estimate, the value is linearly proportional to the solvent-accessible surface area (SASA) because it is basically determined by the interaction with the first layer of solvents. The equation is as follows

(33) $$\Delta G^{0}{}_{solv,nonpolar}=\gamma SASA+b$$

where γ (0.00542 kcal/mol Å) and b (0.92 kcal/mol) are constants fitted to experimental data [383].

The polar solvation energy in implicit solvent $\Delta G^{0}{}_{solv,polar}$ is estimated by Poisson–Boltzmann (PB) model or Generalised Born (GB) model. In the PB model, a solute is represented by an atomic-detail model as in a MM force field, while the solvent molecules and any dissolved electrolyte are treated as a structure-less continuum [308]. The continuum treatment represents the solute as a dielectric body whose shape is defined by atomic coordinates and atomic cavity radii [384]. The electrostatic field can be computed by solving the PB equation: [385]

(34) $$\nabla •\;\left[\varepsilon \left(r\right)\nabla \phi \left(r\right)\right]=-4\pi \rho \left(r\right)-4\pi \lambda \left(r\right)\underset{i}{\sum }z_{i}c_{i}\exp(\frac{-z_{i}\phi \left(r\right)}{K_{B}T})$$

where ε(r) is the dielectric constant, φ(r) is the electrostatic potential, ρ(r) is the solute charge, λ(r) is the Stern layer masking function, $z_{i}$ is the charge of ion type i, $c_{i}$ is the bulk number density of ion type i far from the solute, $K_{B}$ is the Boltzmann constant, and T is the temperature; the summation is over all different ion types. The salt term in the PB equation can be linearised when the Boltzmann factor is close to zero but in highly charged systems the PB equation cannot accurately describe the ionic interactions and correlation enhancement. In such systems, full nonlinear PB equation solvers are more appropriated [308]. The solvation free energy in PB model is represented as [386]

(35) $$\Delta G^{0}{}_{solv,polar}=\frac{1}{2}\sum_{i}q_{i}\;\left[\phi_{\varepsilon =80}\left(r_{i}\right)-\phi_{\varepsilon =1}\left(r_{i}\right)\right]$$

PB is an approach with standard numerical solution, obtaining results of better accuracy. However, the Poisson–Boltzmann equation needs to be solved every time the conformation changes, and hence the computational costs are relatively high in MD application [386].

The GB model is an alternative approach with reasonable approximates and good efficiency. Analytic generalised Born method is used to obtain the estimate of the electrostatic energy of solvation, each atom in molecule is represented as a sphere of radius $R_{i}$ with a charge $q_{i}$ in the centre, dielectric constant ε for solute and solvent are 1 and 80, respectively [294,387]. The equation is as below [381,388]

(36) $$\begin{array}{l} \Delta G^{0}{}_{solv,polar}=-\left(1-\frac{1}{\varepsilon }\right)\underset{i<j}{\sum }\frac{q_{i}q_{j}}{r_{ij}}-\frac{1}{2}\left(1-\frac{1}{\varepsilon }\right)\underset{i}{\sum }\frac{q_{i}{}^{2}}{a_{i}}\\ =-\frac{1}{2}\underset{ij}{\sum }\frac{q_{i}q_{j}}{f_{GB}\left(r_{ij},R_{i},R_{j}\right)}\left(1-\frac{exp\;[-\kappa f_{GB}]}{\varepsilon }\right) \end{array}$$

where $r_{ij}$ is the distance between atoms i and j, the $R_{i}$ are the effective Born radii, and $f_{GB}$() is a certain smooth function of its arguments. The electrostatic screening effects of (monovalent) salt are incorporated via the Debye–Huckel screening parameter κ [308].

The common representation of $f_{GB}\left(r_{ij},R_{i},R_{j}\right)$ is [389]

(37) $$f_{GB}=\sqrt{r^{2}{}_{ji}+R_{i}R_{j}exp(-r^{2}{}_{ij}/4R_{i}R_{j})}$$

The advantages of MM/PB(GB)SA, including desired balance of accuracy/efficiency and the capability of computing absolute binding energy, give this method a much wider use in drug design. Current computational resources allow for the MM/PB(GB)SA to be implemented into the virtual screening workflow as a re-scoring tool to improve the hit rate [390]. MM/PB(GB)SA can also help to investigate the binding free energy of many two-component systems such as protein–ligand [391], protein–protein [350], protein–DNA systems [392] and many more. Moreover, binding free energy decomposition and the contribution of each residue, can be estimated in such a method, which gives key residue-specific information of the binding process [393,394].

## 8. QM/MM and DFT Approaches

Quantum mechanical (QM) and molecular mechanical (MM) calculations can be employed during the drug design process to explore the interaction between ligands and proteins and also how it is processed within the body (ADME). These calculations use a molecular descriptor approach allowing for prediction of ADME properties and modulation in the design process [395]. All of these factors are a consequence of the electronic interactions within a system. The use of molecular mechanics (MM) approaches has been discussed above. Quantum mechanical (QM) approaches provide more realistic results, often in agreement with experimental data, however at significantly greater computational cost when compared to MM [396]. QM approaches can be used to not only study the binding poses but also explore the energy landscape of natural processes or drug-receptor processes [397,398]. QM and MM approaches are used in two main ways to study ligand–protein interactions the first of which only utilises QM to analyse a small region of interest such as the binding site, while the second method also uses QM to analyse the region of interest while using the less computationally expensive MM approach to model the remainder of the system [397,398]. The application of pure Density Functional Theory (DFT) or *ab initio* work is limited due to the expensive computational cost, and as such it is limited to small systems, or for exploring derivable properties [399,400]. However, the application of the hybrid approach allows larger systems to be partitioned with the area of interest (i.e., the active site) being analysed with QM [401]. DFT is a well-established technique, and the experimental design needs to be in line with size, and property being explored for the system. DFT is computationally more efficient and accurate relative to QM (*ab initio*) methods. QM attempts to solve the Schrodinger equation to model the behaviour of the system and this is a non-trivial task for systems where N > 1 (where N is the number of electrons in the system). This can be highly accurate depending on the method employed (i.e., Moller–Plesset vs. Hartree Fock(HF)), however the equation cannot be fully solved as electron correlation effects $\left(E_{XC}\right)$ are unaccounted for. In contrast, DFT explores electronic behaviour of a molecule or system as function of the electronic density, with the energy being directly relatable [402]. This approach allows for much faster generation of a wavefunction to review and the accuracy is dependent on the functional applied. Recently, Bursch et al. provided a thorough review of the functionals and basis set selection for DFT application [403]. Commonly, for DFT in drug design setting, a hybrid functional (Equation (38)) is used

(38) $$E_{XC}^{hybrid}=\alpha \left(E_{X}^{HF}-E_{X}^{GGA}\right)+E_{C}^{GGA}$$

In Equation (38) the α coefficient determines the amount of the exact exchange $\left(E_{X}^{HF}\right)$ derived from first principles that is mixed with the semi-local exchange $\left(E_{X}^{GGA}\right)$. This combined approach was proposed by Becke in 1993, with the first approach being a 50/50 mix for HF and semi-local E_X_ energies [404]. Since, the hybrid functional have grown significantly with an area of HF percentage amounts, commonly, it is between 20–30% [403]. The most commonly applied hybrid is the B3LYP functional containing a scalable 20% HF component [404,405,406]. Alongside a functional is a basis set, which provides numerical functionals for the molecular orbital shape and the occupational. Most commonly a split basis sets such as the 6-311 family is employed [407,408,409,410]. Albeit currently, functionals and basis sets of much higher complexity are being benchmarked and tested (i.e., Coupled-Cluster (CCSD(T), aug-cc-PVDZ, respectively) [411].

The hybrid approaches are more computationally efficient with the trade-off of reduced accuracy in regions away from the active site. However, these methods allow for entire system to be reviewed. QM/MM considers the whole system as conceptually two parts. The active/model region, which uses QM (DFT, commonly). The remaining region is studied using MM (force field approaches), with the boundary between both sites being the QM/MM interactions. The resultant energy of the system takes the form,

(39) $$E_{sys}=E_{QM}+E_{MM}+E_{QM-MM}$$

Here, the system energy ($E_{sys}$) is the summative total of the QM, MM and interface region, respectively. It is obvious that the QM region is more computationally demanding, whilst the peripheries are much more efficient. This approach has been present since the 70s and its impact resulted in a Nobel prize being awarded to Karplus, Levitt and Warshel. [412,413] Since the two-part method, the QM/MM scheme has developed further to the currently more applied, which is our own n-layered Integrated molecular Orbital and Molecular mechanics (ONIOM) and comparative approaches. The ONIOM approach splits a system up to N-layers, with the inner layers closer to the active-site having an electronic density/energy calculated at a higher level of computational theory [401,414]. The MM analyses can be further enhanced by the addition of polarisation terms, solvating the system and even searching for excited states. A thorough review of the ONIOM is provided by Chung et al. and its vast application can be read there [401]. ONIOM has been applied in many computational packages, such as Gaussian and ORCA [415,416]. The boundary selection can be cumbersome, and considering the residue type and possible interactions it produces to influence the level of theory applied [401]. For drug design, ONIOM can be applied to provide energetic information in both structure- and ligand-based approaches. For structure-based approaches, the application of QM or QM/MM can be used to study enzymatic processes, when considering it as an outcome of energy [417,418]. At the core, the understanding of the Michaelis-Menten mechanistic scheme can be used to find rate constants between states [417]. In QM setting this is found by understanding the change in potential energy surface (PES) between states. This approach extends on how ligands interact with targets to understand how the PES is modified or overcome by generation or outcompeting of bonds [400,418,419,420,421]. Extending from PES alone, application analytical tools related to the properties of the wavefunction can be used to describe, modify and improve ligands. The use of frontier molecular orbital (FMO)s can be used to explore the electron donation ability of the ligand by analysis of the HOMO (highest occupied molecular orbital) and LUMO (lowest unoccupied molecular orbital), this can explore priori and postori energy of a ligand on binding [399]. Separately, the interactions present between ligand and receptor can be separated into energetic types to understand how bonding variations occur via R-group selection [399]. Other than ligand interactions and structure-based phenomena, DFT can be utilised to make predictions on binding affinities, pKa, IC_50_, DFT-assisted QSAR, drug-interactions, delivery enhancement and ADME properties [401,414,422,423,424,425,426,427,428,429]. An example of ADME using DFT is the prediction of pKa [430]. DFT was used on the SAMPL6 bind test based on DFT alone and the error ranges were quite large (2–4 pKa units) [430]. Although, when using conceptual DFT (combining molecular descriptors with the DFT results) [431] in a machine learning model, predictions were improved and allowed for extension of the technique to be used for the prediction of non-acidic compounds as well. This approach overall lowered the errors to ~1.85 pKa units [432]. ADME predictions can also be made by utilising global reactivity descriptors, such as the Fukui Functions. This approach allows for the electron density of the molecule to be broken into neutral, positive or negative, which correlates to compounds that can cause electrophilic attack processes to aid in understanding toxicity [432,433]. Although less common in its use, QM/MM approaches have been pivotal in understanding many health burdens, such as, bacteria resistance, and HIV virus proteases process as two examples [422,434,435,436,437,438,439,440,441]. Noting its importance and success in many aspects, DFT or QM/MM approaches although currently under used, are growing in application due to improvement in computation resources. The application of QM or QM/MM can have ample benefit in drug design exploring how and why a process mechanistically occurs.

## 9. Conclusions

Recent advances in computational software and hardware have revolutionised the use of *in silico* methods in drug design, with access to high-performance computers allowing for more complex calculations and larger data sets to be feasibly processed. In this review, we have highlighted a range of *in silico* methods that are commonly used in the hit identification and lead optimisation stages of the drug design process, yet computational methods are also applied in other areas in the pipeline. Some examples include drug repurposing [442,443], protein–protein docking, *de novo* protein design, inverse docking [444], adverse events prediction, physiologically-based pharmacokinetic modelling, and guiding chemical synthesis [442,443].

In addition to classical CADD strategies, such as molecular docking and pharmacophore screening, more accurate and computationally expensive methods, such as MD, DFT, and MM/PB(GB)SA, are now routinely used to further analyse short-listed compounds to better predict binding interactions and docking energies, highlighting compounds which guide us into selecting and optimising the lead compound with the highest success rate.

With the rapid development in artificial intelligence, deep learning-based approaches in drug design have become a trending topic, and various of these strategies were developed for molecular docking [445,446], property prediction [447,448], compound retrosynthesis [449,450,451], *de novo* drug design [452,453] and many more. Although the benefits of incorporating machine learning elements have been highlighted in recent years, there are still certain limitations in these approaches. The training of an algorithm relies heavily on a large amount of data, and therefore the availability of a comprehensive and high-quality dataset directly impacts the performance of the algorithm. Many of the more complex and recent models which utilise machine learning capabilities lack transparency due to their “black box” nature, and the results are not always able to be rationally interpreted and applied, thus limiting the scope of their potential applications in rational drug discovery and design [454]. Nevertheless, the development machine learning based CADD methodologies will be one of the major focuses in the future to continue improving current strategies and to overcome existing challenging barriers in the drug discovery process.

## Data Availability

Not applicable.
